# The Potential for Bouillon Fortification to Reduce Dietary Micronutrient Inadequacy: Modeling Analyses Using National Survey Data from Cameroon, Ghana, and Haiti

**DOI:** 10.1016/j.cdnut.2024.104485

**Published:** 2024-10-18

**Authors:** Reina Engle-Stone, Sika M Kumordzie, Hanqi Luo, Kimberly Ryan Wessells, Seth Adu-Afarwuah, Alex Njebayi, Ismael Teta, Yves-Laurent Régis, Emmanuel Gyimah, Stephen A Vosti, Katherine P Adams

**Affiliations:** 1 Department of Nutrition, University of California, Davis, California, United States; 2 Institute for Global Nutrition, University of California, Davis, California, United States; 3 Department of Global Health, Rollins School of Public Health, Emory University, Atlanta, Georgia, United States; 4 Department of Nutrition and Food Science, University of Ghana, Legon, Accra, Ghana; 5 Helen Keller International, Yaoundé, Cameroon; 6 Partners of the Americas, Port au Prince, Haiti; 7 Department of Agricultural and Resource Economics, University of California, Davis, California, United States

**Keywords:** micronutrient, fortification, bouillon, modeling, dietary adequacy

## Abstract

**Background:**

Bouillon is commonly consumed in some countries where micronutrient deficiencies are prevalent, but it has not been widely adopted as a micronutrient fortification vehicle.

**Objectives:**

We modeled the potential impacts of bouillon fortification on dietary micronutrient adequacy to inform future discussions around bouillon fortification programs.

**Methods:**

We analyzed the dietary intake of women of reproductive age (WRA) and 1- to 5-y-old children from a national dietary survey in Cameroon, and “apparent intake” (using the nutrient density approach) of WRA, children, and men from 3 household surveys in Cameroon, Ghana, and Haiti. We examined (apparent) intake of bouillon and simulated the impacts of bouillon fortification with varying levels of vitamin A, folic acid, vitamin B12, iron, and zinc on inadequate intake (below the estimated average requirement) and intake above the tolerable upper intake level (UL). Scenarios accounted for current mandatory fortification programs and different assumptions about iron absorption from bouillon.

**Results:**

Bouillon was consumed by >67% of households in Ghana and >90% in Haiti and Cameroon. Median (apparent) consumption ranged from 1.6 to 2.1 g/d for WRA, 0.7 to 1.0 g/d for children, and 1.8 to 2.2 g/d for men. Bouillon fortification at the highest micronutrient concentration modeled was predicted to reduce dietary inadequacy by 21–52 percentage points (pp) for vitamin A; 3–47pp for folic acid, and 4–90pp for vitamin B12, depending on the country and population group. In contrast, predicted impacts for iron were modest (2–17pp reduction) but would increase if absorption of iron from bouillon were enhanced. Simulated zinc fortification reduced inadequacy by 12–50pp, but zinc intake above the UL exceeded 10% among children in almost all scenarios.

**Conclusions:**

Modeling indicates that bouillon fortification could improve dietary micronutrient adequacy beyond existing fortification programs. Further work is needed to identify fortification levels that meet criteria for nutritional benefit, technical and commercial feasibility, affordability, and cost-effectiveness.

## Introduction

Micronutrient deficiencies continue to contribute to morbidity and mortality among vulnerable populations globally [1]. Numerous studies have documented the effectiveness of large scale food fortification (LSFF) [2] and their cost-effectiveness relative to alternatives for reducing micronutrient deficiency [[3], [4], [5]]. However, other studies have not found an impact of LSFF on nutritional status [6]; this may be related to aspects of program design, such as selecting an appropriate food vehicle and fortificant [7] or challenges in implementing the program according to plan [4,8]. Food fortification is unlikely to improve population micronutrient status if the food is not commonly consumed by groups at risk for deficiency. Although wheat flour and cooking oil are commonly selected as fortification vehicles, in many settings the reach of “fortifiable” forms of these vehicles is limited [9] and may be disproportionately low among households in rural areas or with low socioeconomic status [10,11]. Other approaches, such as fortification of additional foods, may be needed to reach groups who are vulnerable to micronutrient deficiency but do not typically consume foods that are currently fortified.

Bouillon refers to a flavoring product in a cube, powder, or liquid form that is added to dishes during household cooking. Although fortification of salt with iodine was introduced a century ago, bouillon is a newer candidate condiment for fortification with micronutrients [12,13]. Bouillon is a promising fortification vehicle, particularly in West Africa, where >90% of households in some countries report consuming bouillon regularly, even in rural areas and across socioeconomic strata [11,14]. Furthermore, in many contexts, the bouillon market is dominated by a few major brands that produce the product at large scale; this structure would facilitate efficient monitoring of a bouillon fortification program compared with a market that includes many small- and medium-sized producers. Although the contribution of bouillon products to total sodium intake is an important consideration in light of WHO guidance and global commitments to reduce population sodium intake [15,16], sodium reduction and micronutrient fortification can be complementary efforts [13].

Although to date no countries have adopted fortification standards for bouillon, micronutrients such as iodine, vitamin A, and iron have been added to bouillon products by manufacturers on a voluntary basis [17,18]. Recently, strategies to increase iron bioavailability from fortified bouillon have been tested [[19], [20], [21], [22]], as well as a new technology to maximize vitamin A stability in fortificants [23]. As with other fortified foods, the potential health impacts of fortified bouillon will depend on patterns of consumption of the fortified food (i.e., proportion of typical consumers, and amount consumed) as well as other sources of micronutrients in the diet. However, unlike established food fortification vehicles, there is not currently specific guidance with regard to concentrations of micronutrients to be added to bouillon.

The WHO guidelines for food fortification recommend examining the distribution of micronutrient intakes and the potential contribution of a fortified food to nutrient adequacy to guide decisions about target fortification levels [24]. Ideally, these studies would use nationally representative data on individual intakes and include analyses of subgroups of interest, as illustrated in previous work [25,26]. Where national individual-level dietary data do not exist, alternative approaches and data sources may be employed to inform the selection of fortification levels. In particular, household consumption and expenditure survey (HCES) data have been used to inform the design of LSFF programs [[27], [28], [29]]. However, only a limited number of studies have measured bouillon intake [11,14,30,31] or conducted any modeling to help inform fortification levels [26,32].

Given the potential for micronutrient-fortified bouillon to accelerate progress in reducing micronutrient deficiencies, this study aimed to provide information on bouillon consumption and the potential impacts of bouillon fortification on dietary micronutrient adequacy to inform future discussions around bouillon fortification programs. The specific objectives of the analysis were to (1) estimate bouillon consumption using individual dietary intake or household food consumption data from national surveys in Cameroon, Haiti, and Ghana; (2) assess the adequacy of nutrient intake or apparent nutrient density of the household diet for vitamin A, folate, vitamin B12, iron, and zinc in the same surveys; (3) model the impact of various concentrations of micronutrients added to bouillon on the prevalence of dietary micronutrient inadequacy as well as on intakes above the tolerable upper intake level (UL); and (4) identify possible concentrations of micronutrients added to bouillon that may contribute to achieving dietary nutrient adequacy.

## Methods

### Overview of modeling approach

The general approach to data analysis and modeling was based on that suggested by the WHO [24] for selecting fortification levels. A summary of the data sets used in the analysis is presented in Table 1 [[33], [34], [35], [36]]. The selection of countries was based on (1) expected high prevalence of inadequate micronutrient intake, (2) expected common consumption of bouillon, and (3) data availability. We conducted analyses at the national level, consistent with the level of implementation of LSFF programs, for 5 nutrients: vitamin A, folate, vitamin B12, iron, and zinc.

**TABLE 1:** Characteristics of national surveys included in the analysis.

Survey	Month and year of data collection	Level of data collection	Recall period for food consumption or acquisition data	Sample size	Participant age, y^1^		
					Children	Women	Men
Cameroon 24H [33]	September-December, 2009	Individual	24-hr Recall; replicates in subset	872 Children; 902 Women	2.4 (1.6, 3.2)	26 (22–31)	n/a
Cameroon HH [34]	September-December, 2007	Household	Recall (rural) or diary/notebook (urban) for previous 10 d	11,384 Households	2 (1–3)	27 (21–35)	32 (25–41)
Ghana HH [35]	October 2016–October 2017	Household	Diary with interviews every 3 d^2^ for previous 18 d	11,870 Households	2 (1–3)	29 (22–37)	34 (25–43)
Haiti HH [36]	August–December, 2012	Household	Recall for previous 7 d	4951 Households	2 (1–3)	28 (22–36)	33 (25–43)

1 Median (p25, p75).

2 In households where there was no literate person the interviewer visited the household daily to fill the diary.

Information on individual dietary intake collected by 24-h dietary recall (24hr) was available for this analysis only from Cameroon. For this survey, the prevalence of inadequate intake was defined as the proportion of the target population with nutrient intake below their age- and sex-specific estimated average requirement (EAR) (except for iron, for which the full probability method was used), and the prevalence of high intakes was defined as nutrient intake above the age- and sex-specific UL. We also analyzed data from 3 HCES surveys (Cameroon, Haiti, and Ghana) in which information was collected at the household level, and results for specific target groups within the household were estimated using the nutrient density method [37]. In this method, the average daily micronutrient density of the household diet is calculated as the total nutrient content of the household diet divided by the total energy content of the household diet and expressed as the amount of nutrient per 1000 kcal. This value is then compared with the “critical nutrient density” (CDN) per 1000 kcal for each target group (defined by age, sex, and, where relevant, physiological status), which is calculated as the EAR divided by the estimated energy requirement for that group (except for iron, for which a probability-based method adapted for the nutrient density method is used [38]. The interpretation of adequate nutrient density is that the household diet is adequate to meet nutrient needs of a particular target group if they are meeting their energy requirements and food is distributed among household members in proportion to their age- and sex-specific energy requirements. Although we are not aware of a formal validation of this approach (i.e., comparing the nutrient density method applied to HCES data to individual-level assessment of dietary adequacy in relation to the EAR), previous studies reported similar nutrient density estimated from HCES compared with 24-hr data [39], and mixed results for comparison of the nutrient density approach compared with the EAR cut-point approach applied to 24-hr data [37]. The nutrient density method was selected to assess nutrient adequacy for HCES data in the current analysis as it may be less sensitive to mis-estimation of the total quantity of food consumed by the household [29], and the approach has now been adopted by many analysts working with HCES data [38,40,41]. Other comparisons of 24hr and household survey data suggest that use of household-level data to make inferences about individual dietary adequacy is reasonable for adults [29], but household data are less accurate for young breastfeeding children in particular [42]. For simplicity we refer to “inadequate intake” throughout the article but we note that the results based on analysis of HCES data are more accurately described as “inadequate apparent household nutrient density.”

### Data sources

#### Cameroon national micronutrient survey, 2009

The Cameroon National Micronutrient Survey, conducted in 2009, was a national multistage cluster survey with 3 strata: the North, South, and Yaoundé/Douala [14,33]. Eligible households included a woman of reproductive age and 12- to 59-mo-old children. At recruitment, interviewers administered 24hr in a subset of participants. When households were visited 2 d later for additional data collection, interviewers administered 24hr to all participants to collect information on dietary intake among women (*n* = 912) and children (*n* = 883). The 24hr interview followed a multipass interview method [43]. Interviews included collection of recipe data (including types and quantities of ingredients, and total quantity prepared) from respondents who had prepared the mixed dishes they reported consuming. For mixed dishes containing meat that was not evenly distributed in the dish (e.g., several large chunks of meat compared with dried fish powder), the enumerator recorded separately the respondent’s portion of the meat consumed. Additional details on data collection methods and study results have been described previously [26,44]. The study was approved by the Cameroon National Ethics Committee and the Institutional Review Board of the University of California, Davis.

#### Third Cameroonian household survey, 2007

Data were collected for the Third Cameroonian Household Survey (Troisième Enquête Camerounaise auprès des Ménages, ECAM3) between September and December 2007. The sampling design for the nationally representative survey, which was conducted by the Cameroon National Institute of Statistics, was a 2-stage stratified random sample with 32 strata; each of the 10 regions of Cameroon was divided into 3 strata (urban, semi-urban, and rural) and the 2 main cities, Yaoundé and Douala, were treated as separate stratum [34]. For rural households, data on household food expenditures and acquisitions were collected twice. During the first visit, households were asked to recall all food acquired over the past 7 d. Four days later, rural households were visited again and asked about daily food acquisitions on each of the previous 3 d. Urban and semi-urban households were given a notebook to record their food acquisitions, and these households were visited approximately every 3 d for a period of 18 d to collect data from their notebooks. Because rural households had a maximum of 10 d of recall, whereas urban and semi-urban households had 15 d of recall, we used the first 10 d of recall from all households to make the number of days of recall included in the analysis equal across all households.

Results for both surveys from Cameroon were included because the 24hr is expected to be more accurate for estimating individual intake, but the household survey provides estimates of household food consumption over a longer period of recall and allows for estimates of the potential impact of bouillon fortification among men. Previous work evaluated detailed comparisons between the 2 data sets for (1) intake of fortifiable foods [45] and (2) estimated micronutrient intake and predicted impact of various micronutrient interventions [32]. Although there are some differences in prevalence estimates for inadequate nutrient intake between the 2 data sets, the results were similar with regard to subnational patterns of inadequate intake and relative rankings of the predicted impact of LSFF programs on dietary adequacy.

#### Haiti survey on household living conditions after the earthquake, 2012

For Haiti, apparent food consumption and nutrient densities of household diets were based on the 2012 National Household Living Conditions Survey after the Earthquake (Enquête sur les Conditions de Vie des Ménages Après Seism, ECVMAS). The 2012 ECVMAS was conducted between August and December by the Haitian Institute of Statistics and Information (IHSI) using a 2-stage stratified cluster sample designed to be representative at the level of Haiti’s 10 administrative units (départements) as well as nationally [35]. The survey asked household respondents to report the quantity of food consumed by the household in the 7 d preceding the survey.

#### Ghana living standards survey (GLSS7), 2016–2017

The Ghana Living Standards Survey (GLSS) is a nationally representative household survey conducted by the Ghana Statistical Service (GSS) [36]. The seventh round of the survey, GLSS7, conducted over a 12-mo period between October 2016 and October 2017, employed a 2-stage stratified sampling design. One thousand enumeration areas were selected to form the primary sampling units (PSUs). The PSUs were allocated into the 10 administrative regions using a probability proportional to population size approach. The list of enumeration areas from which the samples were drawn was based on the 2010 Population and Housing Census. The enumeration areas were further divided into urban and rural localities of residence. A complete listing of households in the selected PSUs was undertaken to form the Secondary Sampling Units. At the second stage, 15 households from each PSU were systematically selected. The total sample size came to 15,000 households nationwide. Survey participants were provided with a diary to record daily food expenditures over 18 d and in addition were visited every 3 d for interviews during the period. For households without a literate person, enumerators visited the households daily to record the daily food expenditures in the diary [36].

### Nutrient intake estimation: 24-hour dietary recall

Nutrient intakes were calculated using a food composition table based primarily on the Nutrient Data System for Research (NDSR; Nutrition Coordinating Center, University of Minnesota) [46], with additional values drawn from HarvestPlus food composition table for Uganda, the USDA food composition database [47], the International Minilist, and published literature, as described previously [44]. Household recipes were used to construct generic recipes for mixed dishes for which recipes were not available, by first computing the nutrient quantity per unit volume (e.g., mg iron per 100 mL recipe) for each household recipe collected, and then averaging these values by type of mixed dish.

For the 24-h dietary recall data only, vitamin B12 absorption was estimated using a modified version of the prediction equation published by Doets et al., consistent with previous analyses [48,49] (Supplemental Table 1), and iron absorption from the total diet, excluding bouillon, was estimated using a mathematical prediction equation for absorption of total daily nonheme iron [50]. Due to data availability, only plasma ferritin concentrations (measured among the same individuals who participated in the dietary survey) and phytate intake (assessed in the dietary survey using phytate data from the NDSR [46]) were used in the prediction of iron absorption [48]. To consider the contribution of heme iron, we assumed that 10% of iron was consumed as heme iron [51], and that 25% of heme iron was absorbed [51]. Absorbable zinc was estimated using published equations for adults [52] and children [53].

The National Cancer Institute (NCI) method is a well-established method of analysis for foods and nutrients using 24-h dietary data. It utilizes freely available SAS macros, *MIXTRAN* and *DISTRIB*, to remove within-person variation and estimate long-term usual intake distributions and prevalence of inadequate nutrient intake. We used the SIMPLE macro [54], which integrates the existing NCI *MIXTRAN* and *DISTRIB* macros to estimate usual intake distributions, to estimate mean and percentiles of intake of bouillon and each nutrient of interest, as well as prevalence of inadequate intake and intake above the UL; we applied the balanced repeated replication method to calculate standard errors. For breastfed children, we also included nutrient intake from human milk in estimating prevalence of inadequate nutrient intake by the “shrink-then-add” method [55], by first applying the NCI method and then adding the estimated daily nutrient intake from human milk (calculations shown in Supplemental Table 2). The original data set included 12- to 59-mo-old children; we extrapolated the results to represent the population of 6- to 59-mo-old children by poststratification based on breastfeeding status [56].

### Nutrient intake estimation: household consumption and expenditure surveys

Household reported quantities of food were converted to grams and, where applicable, the total quantity was adjusted for the edible portion and the yield factor from cooking. No adjustments were made for waste or food loss, and it was assumed that all food acquired by the household was consumed by household members during the recall period. The daily average of the total quantity of each food acquired during the recall period of each survey was used as a proxy for usual household consumption, or “apparent” consumption. Nutrient densities were then estimated from food composition tables. For Cameroon and Haiti, foods were primarily matched with entries from the NDSR database [46], supplemented with entries from the West African food composition table [57] and USDA food composition database [47]. For Ghana, foods were primarily matched with entries from the West African food composition table and supplemented with entries from the NDSR and USDA databases.

Iron absorption from all dietary sources except fortified bouillon was assumed to be 10% based on the dietary patterns represented in these countries [24] (the algorithm for nonheme iron absorption could not be applied to HCES data because information on ferritin concentration is not available from these surveys). As above, absorbable zinc was estimated using published algorithms for adults [52] and children [53] (Supplemental Table 1).

Given the household-level reporting of food expenditures and consumption in the HCES data, we used the adult male equivalent (AME) method to estimate individual-level bouillon consumption from the household-level apparent food consumption [58]. This method assumes the intrahousehold distribution of food is proportional to each household member’s age- and sex-specific energy requirements. Using household composition data collected as part of each HCES, we calculated an AME weight for each household member as the ratio of the household member’s energy requirements to the energy requirements of a male age 18–30 y, assuming moderate physical activity levels [59]. Because maternity status was not identified in the household composition data, AME weights for all women of reproductive age (WRA) were based on the energy requirements of nonpregnant, nonlactating women. Before analysis, extreme outliers in apparent consumption of each food in the food list, defined as apparent consumption per AME per day above the 95th percentile of apparent consumption per AME per day, were replaced with the 95th percentile value.

We calculated the nutrient density of household diets as the total average daily apparent nutrient intake divided by the total average daily apparent energy intake, expressed per 1000 kcal by multiplying that ratio by 1000. For each of the 3 target populations of interest (6- to 59-mo-old children, WRA, and adult men), we also calculated a “critical nutrient density,” which is the amount of nutrients per 1000 kcal that would be required to meet nutrient requirements for a given age/sex group [33]. The critical nutrient density was calculated as the ratio of the age- and sex-specific EAR to age- and sex-specific energy requirements, multiplied by 1,000, where energy requirements were estimated relative to the FAO human energy requirement estimate of 2,900 kcals for an 65kg adult male age 18-30 years with moderate physical activity [59].

Finally, if a household had >1 member in that target group, we randomly selected one household member to include in analyses of that target group. Similarly, if a household did not have a member in a specific target group, that household was not included in analyses of that target group.

### Data analysis

For all surveys, we conducted descriptive analyses using appropriate survey weights to summarize population characteristics, calculate the proportion of individuals or households that consumed bouillon during the study period, and estimate usual bouillon consumption. Analyses were conducted at the national level, reflecting the level of LSFF program implementation.

For all nutrients except iron, we calculated the prevalence of inadequate intake (or apparent intake) using the EAR cut-point method (24-hr survey) or the nutrient density method (household surveys). For iron, the full probability approach was applied to the Cameroon 24hr data (Supplemental Table 1) and a modified version of the full probability approach adapted for assessing iron densities was applied for the household data [37]. Prevalence of high intakes was defined as that exceeding the UL in the 24-hr data (Supplemental Table 2), or nutrient density exceeding the tolerable upper nutrient density (calculated as the ratio of the age- and sex-specific UL to age- and sex-specific energy requirements, multiplied by 1000) for household surveys. Evaluation of intakes above the UL was restricted to preformed retinol for vitamin A and to folic acid for folate; no UL has been defined for vitamin B12 [60,61]. EAR and UL values were from the United States Institute of Medicine, with the exception of zinc, for which physiological requirements from the European Food Safety Authority (EFSA) were used for children and corrected International Zinc Nutrition Consultative Group (IZiNCG) values were applied for adults [62] (Supplemental Tables 3 and 4). Reference values were matched to the characteristics of each observation in the data set (i.e., by age and sex, and, for 24hr only, whether women were pregnant or lactating).

Each of the countries included in this analysis has legislation regarding mandatory fortification of staple foods such as cooking oil and wheat flour, although information on program implementation was variable. The primary analysis represented the “status quo” for current programs: we accounted for the estimated current contribution of these fortified staple products to nutrient intakes using values for the micronutrient content of each food from published literature, where available, and estimates from in-country key informants otherwise (Table 2) [[63], [64], [65]]. For this calculation we did not distinguish between partial fortification and nonfortification; the values for “actual” nutrient content (representing an average value across all fortifiable products) were applied to 100% of each fortifiable product. As a sensitivity analysis, we also examined the contribution of fortified bouillon in alternative scenarios assuming that any foods subject to mandatory fortification programs are fortified at the target micronutrient levels.

**TABLE 2:** Target micronutrient content of mandatory large scale fortification programs in selected countries, and estimated micronutrient content under program conditions.

		Cooking oil	Wheat flour				
		Vitamin A, mg/kg	Vitamin A, mg/kg	Folic acid, mg/kg	Vitamin B12, mg/kg	Iron, mg/kg	Zinc, mg/kg
Cameroon	Target	12	n/a	5	0.04	60	95
	Actual^1^	9	n/a	1.65	0.013	19.8	31.35
Haiti	Target	15.02	n/a	1.5	n/a	30	60
	Actual^2^	12.02	n/a	0.57	n/a	11.4	22.8
Ghana	Target	10.0	2.0	2.08	0.01	58.5	28.3
	Actual^3^	10.6	0.8	0.81	0.004	22.8	11.0

1 Fortification levels in Cameroon assumed to be 75% of target for oil and 33% of target for wheat flour [63]; the proportion of products that were not fortified compared with partially fortified was not available, but 38% of oil samples and 7% of wheat flour samples had micronutrient content within the range given in the national standard.

2 For Haiti, wheat flour was assumed to be fortified at 38% of target levels, and oil was assumed to be fortified at 80% of target levels. The assumption for wheat flour is roughly consistent, although a slight underestimate, compared with a recent report that estimated that 48% of wheat flour in Haiti was fortified at or above the target minimum level for iron, 12% was partially fortified, and 40% was not fortified [64]. Similarly, the assumption for oil is roughly consistent with the report’s estimate that 73% of market share was fortified at or above the target minimum for vitamin A and 27% was not fortified.

3 Fortification levels in Ghana assumed to be 106% of target for oil and 39% of target for all micronutrients wheat flour, based on analysis of oil vitamin A content and wheat flour iron content in the Ghana Micronutrient Survey [65]. In the same survey, 55.6% of oil samples were found to be “adequately fortified” (>10 ppm) and 5.7% of wheat flour samples were “adequately fortified” with iron (≥58.5 ppm).

### Simulations of the impact of bouillon fortification on nutrient intake or nutrient density

To simulate the impact of bouillon fortification on nutrient intake using the 24hr data, bouillon consumption (grams per day) for each individual observation was multiplied by the selected fortification level to calculate the amount of nutrient provided by bouillon. This total was added to the “baseline” micronutrient intake from other food sources, including consumption of other fortified foods, for each individual (analogous to the “add then shrink” approach for 24hr analysis [55, 66]). Using household data, the contribution of fortified bouillon to the nutrient density of the household diet was calculated by multiplying the total daily apparent household bouillon consumption by the selected fortification level, and then that value was divided by total daily apparent household energy intake and multiplied by 1000. Then, to model the impact of bouillon fortification, that value was added to the “baseline” nutrient density of the household diet. For each scenario, we then recalculated the prevalence of inadequate and high intakes/densities. Although some brands of bouillon currently contain added iron or vitamin A, for simplicity and in the absence of detailed information on bouillon consumption by brand, we did not include the contribution of this voluntary fortification in the analysis.

We also did not account for micronutrient supplement use or other interventions such as micronutrient powders. Information on micronutrient supplement use was collected in the Cameroon 24hr survey but was not used due to the low reported frequency and lack of detail on nutrient content of supplements. This information was not collected in the household surveys, but data from the Ghana Micronutrient Survey indicated that only a small proportion of children had received micronutrient powders (1.5%) or ready-to-use therapeutic foods (1.1%) in the previous day, although 22.5% of children were reported given iron tablets or syrup in the past 6 mo and 20.7% of children were reportedly given multivitamins the past 6 mo [63], so the results may slightly underestimate dietary adequacy for some nutrients. Similar coverage data were not available for Haiti at the time of the survey. All 3 countries have programs to distribute high-dose vitamin A supplements, and we did not account for those here because coverage may vary annually and because the supplementation programs could theoretically be adjusted in response to changes in fortification programs more easily than the reverse. That is, if fortification is successful in reducing dietary inadequacy, then supplementation programs might not be necessary, but planning fortification programs assuming a constant coverage of high-dose vitamin A supplements does not match the reality of annual variation in supplement coverage and ignores the potential for more efficient supplement targeting if successful fortification programs are in place.

The fortification levels tested ranged from 20 to 250 μg vitamin A per gram bouillon, 20 to 120 μg folic acid per gram bouillon, 0.2 to 2.0 μg vitamin B12 per gram bouillon, 0.6 to 5 mg iron per gram bouillon, and 0.6 to 5 mg zinc per gram bouillon (Supplemental Table 5). In each scenario, we assumed that 100% of bouillon consumed was fortified at the selected concentration. Although in practice many factors may hinder the ability to fortify 100% of a product, this assumption was chosen to represent the “best case scenario” for fortification at a given nutrient concentration; examination of lower fortification levels could provide a rough estimate of the potential effects on dietary adequacy in the case of lower compliance. Similarly, for the purpose of this analysis, the nutrient concentrations modeled reflect the concentration at the time of consumption, without factoring in further assumptions about nutrient retention.

For the primary analysis, we assumed that 2% of the iron from fortified bouillon would be absorbed [19,21]. However, we conducted sensitivity analyses assuming 4% and 10% iron absorption to explore the potential impact of fortification with more bioavailable forms of iron and/or consumption of iron-fortified bouillon among populations with depleted iron stores [20,22]. For zinc, the additional zinc from bouillon was added to dietary zinc from other sources before applying the absorption algorithms referenced above; the same approach was used for absorbable vitamin B12 in the 24hr analysis.

### Criteria for selecting ranges of fortification levels

For each survey, we identified the range of fortification levels, among the levels modeled, that would yield the greatest decrease in adequate intake or apparent consumption while maintaining estimates of high intake below a selected threshold for all groups, i.e., the general approach suggested in the WHO fortification guidelines: “The amount of micronutrient added to the diet through food fortification should be designed such that the predicted probability of inadequate intakes of that specific nutrient is <2.5% for population subgroups of concern, while avoiding risk of excessive intakes in other subgroups of the population” [24]. For zinc, given the uncertainty around the UL for preschool children [67], we conducted this exercise with and without consideration of children’s intakes above the UL. We used 2 different approaches to exclude fortification levels as “too high,” based on (1) point estimates for prevalence of intake above the UL ≥ 0.5%, ≥ 5.0%, or ≥ 10.0%, or (2) lower 95% confidence interval of the prevalence of intake above the UL ≥ 0.5%, ≥ 5.0%, or ≥ 10.0%. Because the modeling evaluated a discrete set of 7 fortification levels for each micronutrient (Supplemental Table 5), the results do not necessarily represent the “optimal” level for a given country; however, this approach allowed us to identify a range of fortification levels that potentially meet criteria set out by the WHO guidelines.

## Results

### Population characteristics and bouillon consumption

Median age of participants in the different countries was similar and ranged from 2.0 to 2.4 y for children, 26 to 29 y for women, and 32 to 34 y for men. In the Cameroon 24hr survey, 21% of children were currently breastfed; information on breastfeeding status was not available from the household surveys (Table 1).

Bouillon was commonly consumed in all countries included in the analysis. In the 24hr survey, over 90% of women and children in Cameroon reported bouillon consumption in the previous day, whereas any consumption during the recall period estimated from the household surveys ranged from 71% of households in Ghana (67%–81%, depending on household composition) to over 90% in Haiti (Table 3) [54,58]. Median apparent bouillon consumption across all countries ranged from 1.6 to 2.1 g/d for WRA, 0.7–1.0 g/d for children, and 1.8–2.2 g/d for men.

**TABLE 3:** Bouillon consumption patterns among women, children, and men, by survey.

		Proportion consuming (or acquiring)^1^	Daily bouillon intake^2^, g/d	
		% (95% CI)	Mean (95% CI)	Median (p25, p75)
Women of reproductive age				
	Cameroon 24H	93.0 (90.1, 95.5)	2.1 (1.7, 2.5)	1.9 (1.2, 2.7)
	Cameroon HH	89.3 (88.3, 90.2)	1.8 (1.7, 1.8)	1.6 (0.8, 2.6)
	Haiti HH	96.6 (95.8, 97.3)	2.2 (2.2 - 2.4)	1.7 (1.0, 2.9)
	Ghana HH	75.8 (74.5, 77.0)	2.4 (2.3, 2.4)	2.1 (0.6, 4.3)
Children 6–59 mo of age				
	Cameroon 24H	93.0 (90.1, 95.5)	1.0 (1.0, 1.0)	1.0 (0.6, 1.3)
	Cameroon HH	89.3 (88.0, 90.6)	0.7 (0.7, 0.8)	0.7 (0.3, 1.1)
	Haiti HH	97.5 (96.6, 98.2)	1.0 (1.0, 1.1)	0.8 (0.4, 1.3)
	Ghana HH	80.5 (78.8, 82.1)	1.1 (1.1, 1.2)	1.0 (0.4, 2.0)
Adult men				
	Cameroon HH	83.1 (82.0, 85.2)	2.0 (2.0, 2.1)	1.8 (0.7, 3.2)
	Haiti HH	95.2 (94.3, 96.0)	2.8 (2.7, 2.9)	2.0 (1.2, 3.5)
	Ghana HH	66.7 (65.3, 68.0)	2.6 (2.5, 2.6)	2.2 (0.0, 4.9)

1 Corresponding data collection periods are: Cameroon-24hr: past 24 h; Cameroon-HH, past 10 d; Ghana HH: past 18 d; Haiti HH: past 7 d. 24hr, 24-hour dietary recall; HH, household survey. Estimates represent only households that include ≥1 person in the selected age- and sex-specific category and thus the proportion consuming may vary across target groups within a survey. The estimates of bouillon consumption based on household survey data assume that food is distributed within the household in proportion to each member’s age- and sex-specific energy requirements. While the 95% confidence intervals presented here represent the statistical uncertainty based on the dataset analyzed, they do not capture uncertainty in the intrahousehold distribution of food or other limitations of using household consumption and expenditure survey (HCES) data for estimating dietary intake

2 Calculated using all available days of data. Apparent intake refers to individual consumption estimated from household data by the adult male equivalent method [33]. For Cameroon 24H, usual intake distributions estimated using the SIMPLE macro [56].

### Adequacy of micronutrient intake and micronutrient density

**FIGURE 1:**
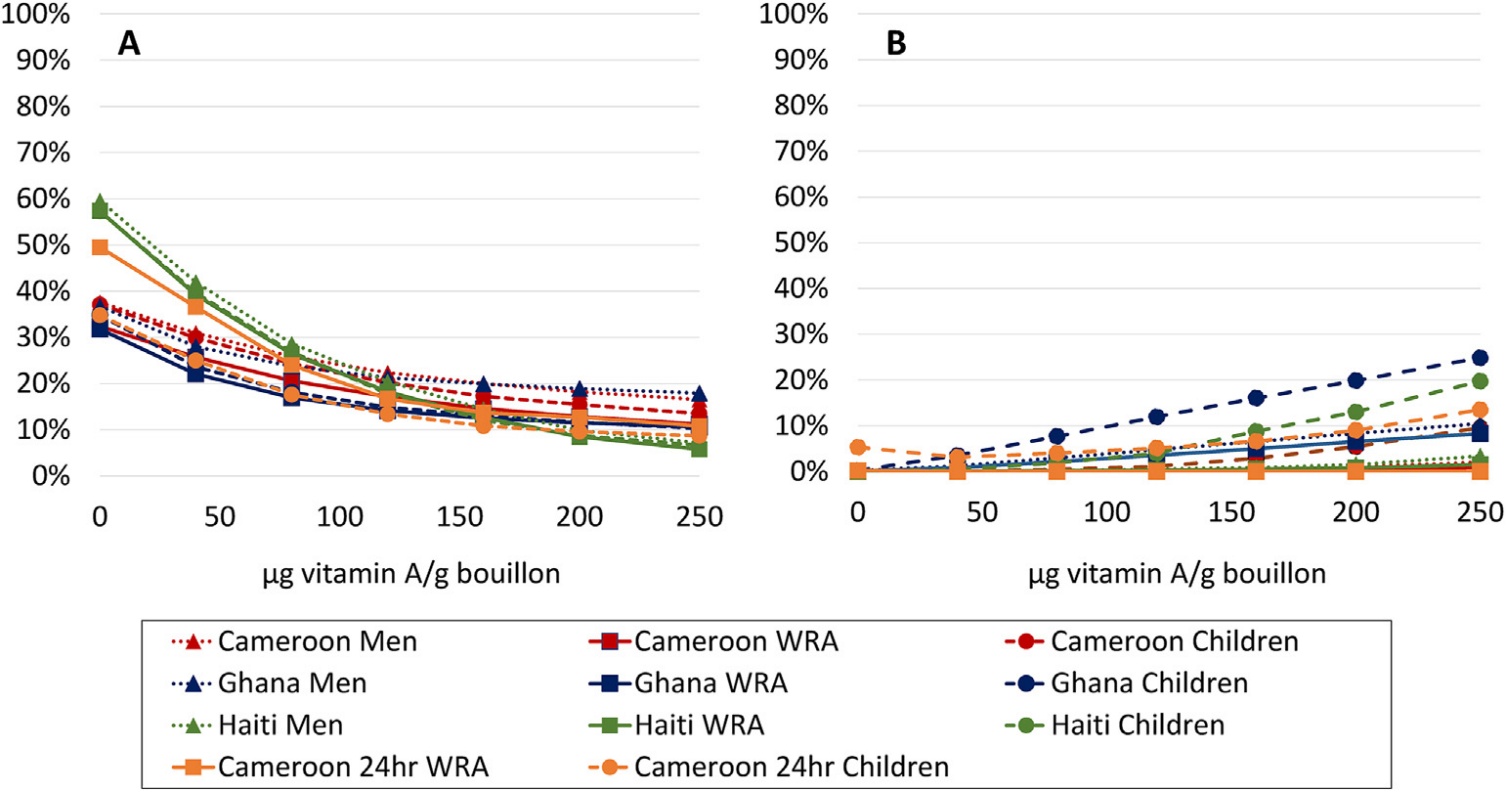
Prevalence of (A) inadequate or (B) high intake (Cameroon 24-h dietary recall) or apparent nutrient densities (all other data sets) of vitamin A among women of reproductive age, men, and 6- to 59-mo-old children at different modeled levels of vitamin A added to bouillon. For 24-h dietary recall data, inadequate intake was defined as total vitamin A (expressed as μg retinol activity equivalents [RAE]/d) below the age- and sex-specific estimated average requirement, whereas high intake was defined as preformed retinol (expressed as μg/d) above the tolerable upper intake level. For household consumption and expenditure survey data, inadequate apparent nutrient density was defined as vitamin A density of the household diet below the age- and sex-specific critical nutrient density, and high apparent nutrient density was defined as retinol density of the household diet above the age- and sex-specific tolerable upper density. Simulations account for fortification of cooking oil (9 mg/kg in oil for Cameroon; 12.02 mg/kg Haiti; 10.6 mg/kg Ghana) and wheat flour (0.8 mg/kg for Ghana) at estimated current levels of compliance with fortification standards. Technical considerations may limit the feasibility of certain fortification levels; concentrations ≤200 μg/g have been successfully added to bouillon in a research context [68].

**FIGURE 2:**
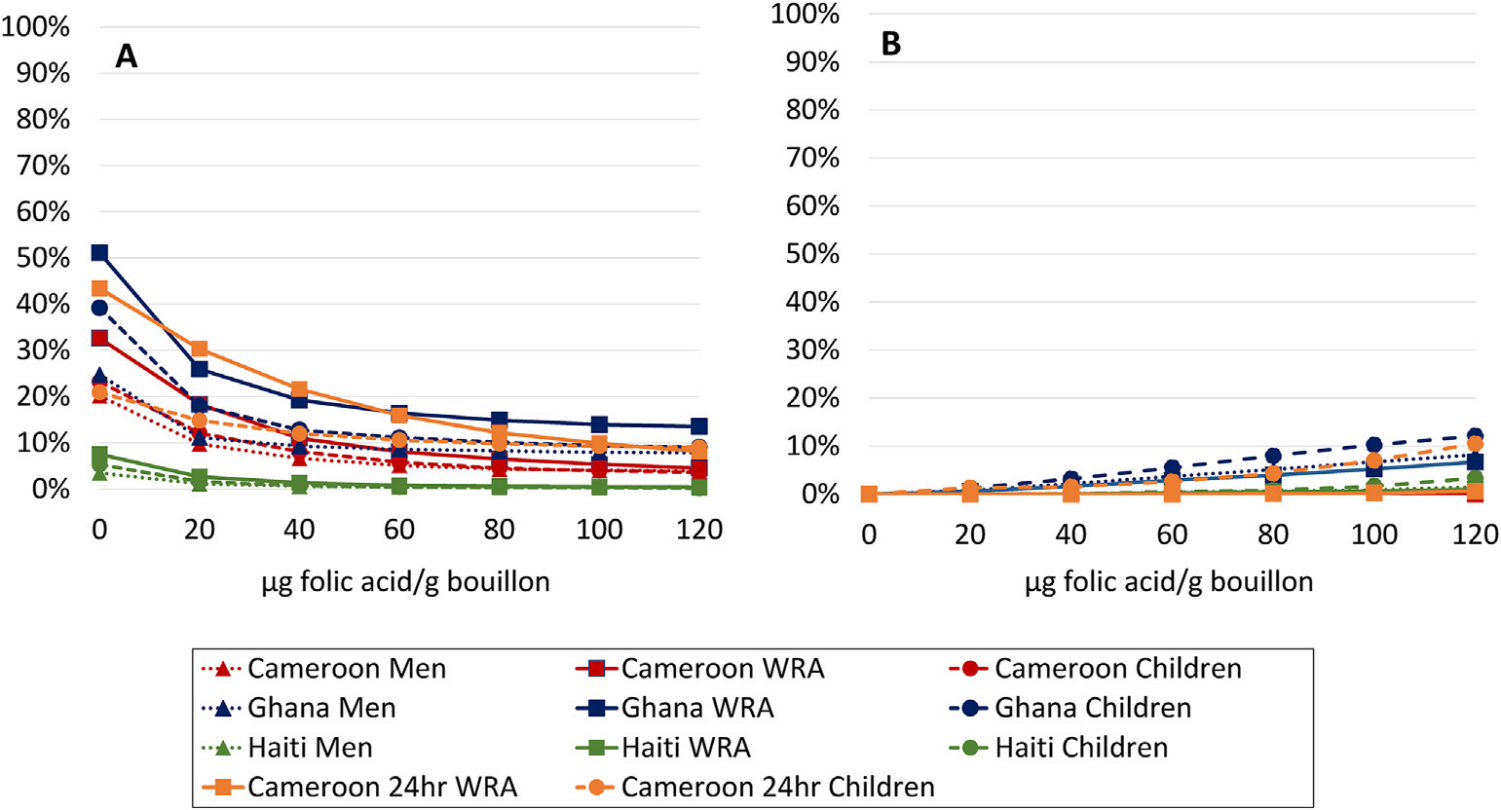
Prevalence of (A) inadequate and (B) high intake (Cameroon 24hr) or apparent nutrient densities (all others) of folate among women of reproductive age, men, and 6- to 59-mo-old children at different modeled levels of folic acid added to bouillon. For 24-h dietary recall data, inadequate intake was defined as total folate (expressed as μg dietary folate equivalents [DFE]/d) below the age- and sex-specific estimated average requirement, whereas high intake was defined as folic acid (expressed as μg/d) above the tolerable upper intake level. For household consumption and expenditure survey data, inadequate apparent nutrient density was defined as folate density of the household diet below the age- and sex-specific critical nutrient density, and high apparent nutrient density was defined as folic acid density of the household diet above the age- and sex-specific tolerable upper density. Simulations account for fortification of wheat flour at estimated current levels of compliance with fortification standards (1.65 mg/kg in wheat flour for Cameroon; 0.57 mg/kg Haiti; 0.81 mg/kg Ghana). Technical considerations may limit the feasibility of certain fortification levels; concentrations ≤80 μg/g have been successfully added to bouillon in a research context [68].

**FIGURE 3:**
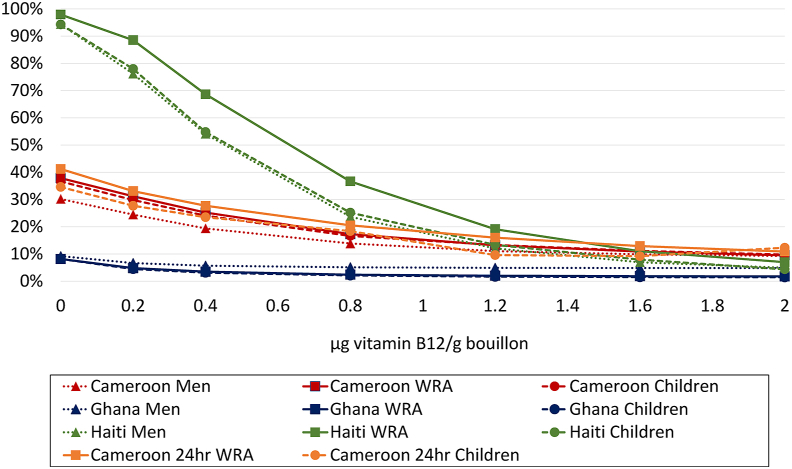
Prevalence of inadequate intake (Cameroon 24hr) or apparent nutrient densities (all others) of vitamin B12 among women of reproductive age, men, and 6- to 59-mo-old children at different modeled levels of vitamin B12 added to bouillon. For 24-h dietary recall data, inadequate intake was defined as vitamin B12 (expressed as μg/d) below the age- and sex-specific estimated average requirement. For household consumption and expenditure survey data, inadequate apparent nutrient density was defined as vitamin B12 density of the household diet below the age- and sex-specific critical nutrient density. High intakes were not modeled because no tolerable upper intake level has been defined for vitamin B12. Simulations account for fortification of wheat flour at estimated current levels (0.013 mg/kg in wheat flour for Cameroon; n/a Haiti; 0.004 mg/kg Ghana). Technical considerations may limit the feasibility of certain fortification levels; concentrations ≤1.2 μg/g have been successfully added to bouillon in a research context [68].

**FIGURE 4:**
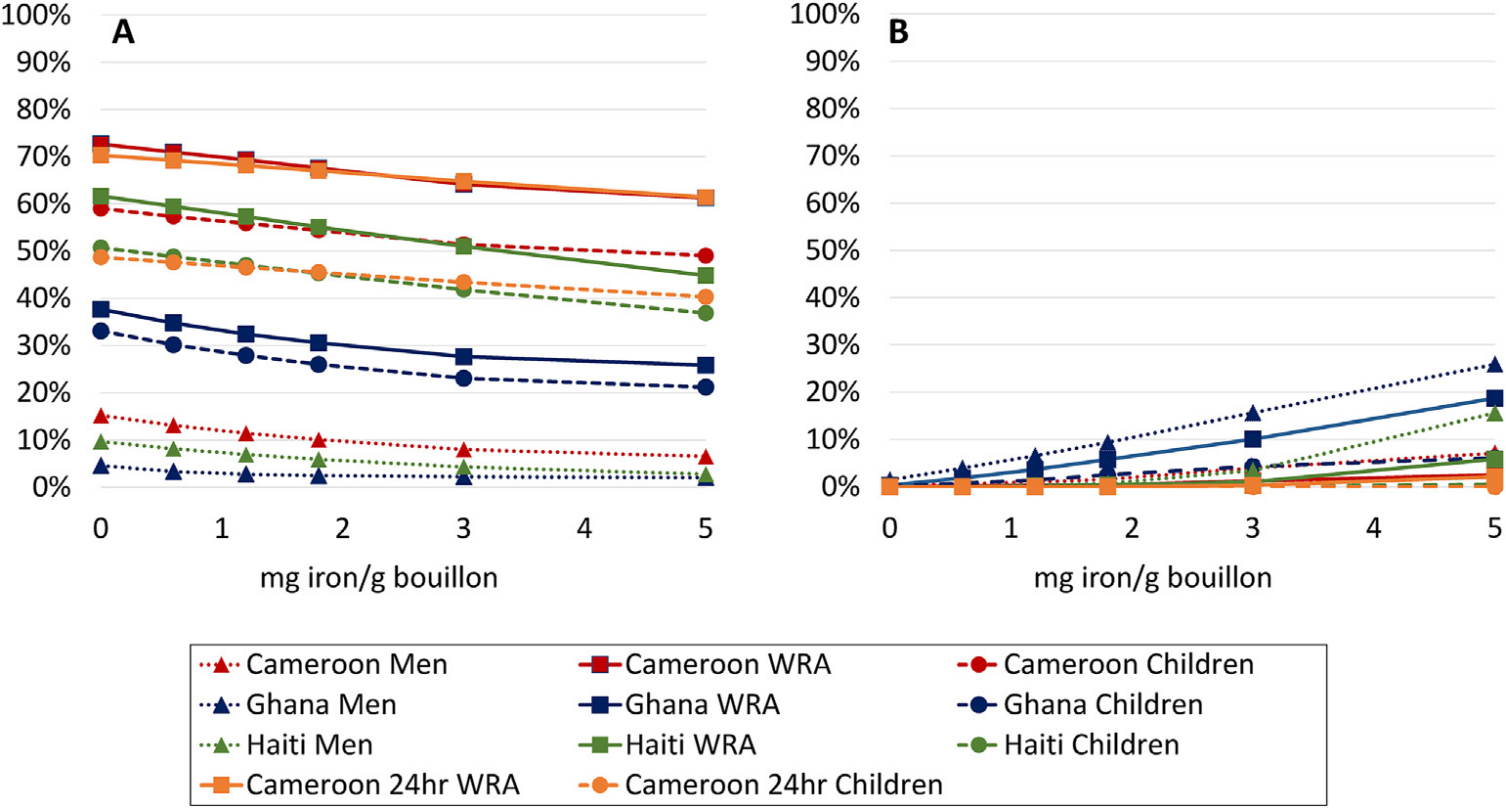
Prevalence of (A) inadequate and (B) high intake (Cameroon 24hr) or apparent nutrient densities (all others) of iron among women of reproductive age, men, and 6- to 59-mo-old children at different modeled levels of iron added to bouillon. Inadequate intake was calculated using the full probability approach as inadequate absorbable iron intake (24 h dietary recall data) or iron density of the household diet (household consumption and expenditure survey). Nonheme iron absorption was estimated according to Armah et al. [50] for the Cameroon 24-h recall data set (see Methods for details); in all other surveys, iron absorption was assumed to be 10% from the total diet. Iron absorption from fortified bouillon was assumed to be 2%. Simulations account for fortification of wheat flour at estimated current levels (19.8 mg/kg in wheat flour for Cameroon; 11.4 mg/kg Haiti; 22.8 mg/kg Ghana). Technical considerations may limit the feasibility of certain fortification levels; concentrations ≤4 mg/g have been successfully added to bouillon in a research context [68].

**FIGURE 5:**
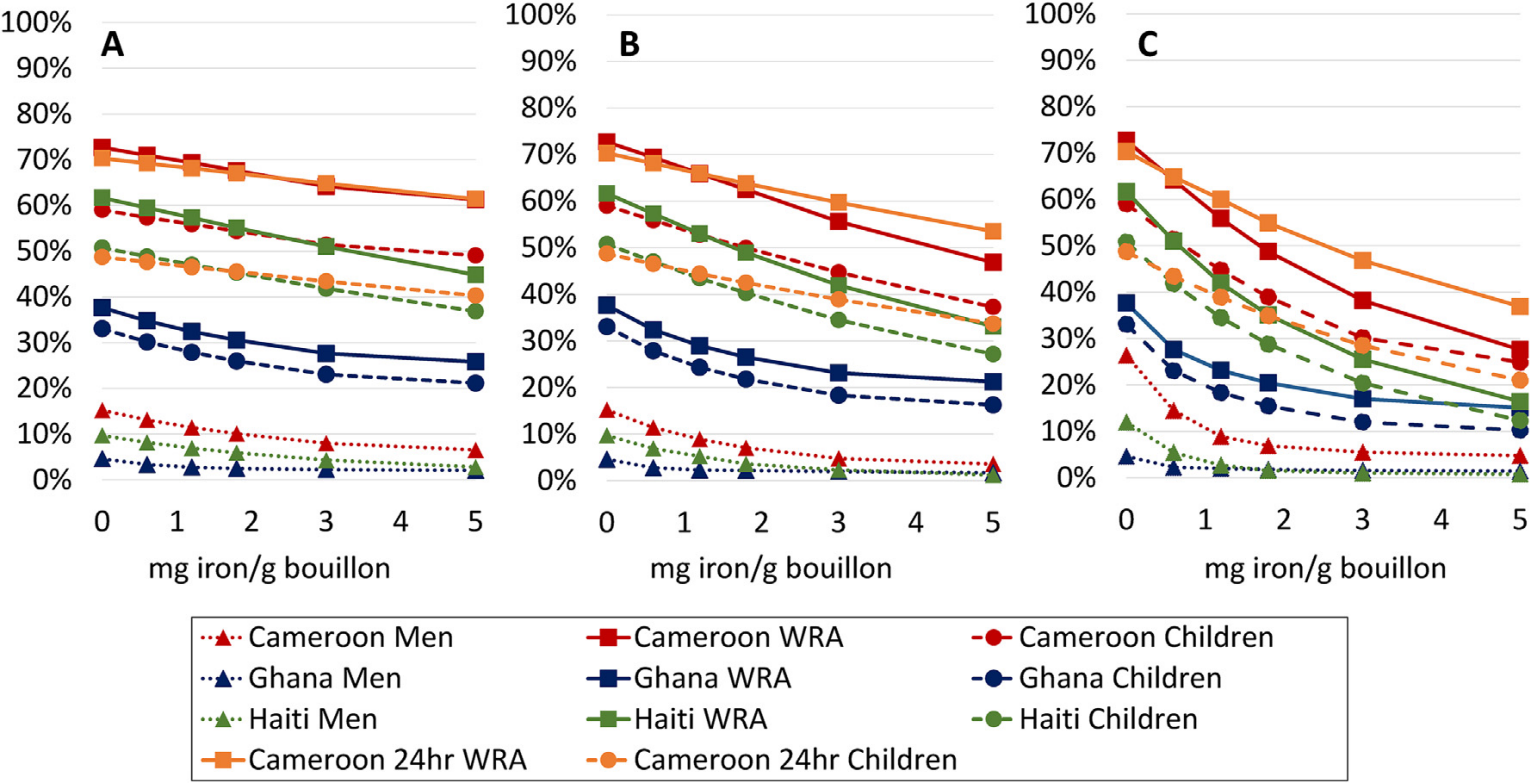
Prevalence of inadequate intake (Cameroon 24hr) or apparent nutrient densities (all others) of iron among women of reproductive age, men, and 6- to 59-mo-old children at different assumed levels of iron added to bouillon, and assuming either 2% (A), 4% (B), or 10% (C) absorption of iron from bouillon. Inadequate intake was calculated using the full probability approach as inadequate absorbable iron intake (24 h dietary recall data) or iron density of the household diet (household consumption and expenditure survey). Nonheme iron absorption from dietary sources other than bouillon was estimated according to Armah et al. [50] for the Cameroon 24-h recall data set and assumed to be 10% from the total diet for all other sources (see Methods for details). Simulations account for fortification of wheat flour at estimated current levels (19.8 mg/kg in wheat flour for Cameroon; 11.4 mg/kg Haiti; 22.8 mg/kg Ghana).

In the presence of “status quo” large scale fortification programs (i.e., at current levels of compliance with published standards), micronutrient inadequacy generally exceeded 30%, except for folate among all groups in Haiti (3%–8%) among children and men in Cameroon (20%–23%), and among men in Ghana (25%), vitamin B12 among all groups in Ghana (8%–9%), and zinc among women in Haiti (27%) (FIGURE 1, FIGURE 2, FIGURE 3, FIGURE 4, FIGURE 5 [68]; Supplemental Tables 6 and 7). For iron, dietary inadequacy was low among men, ranging from 5% in Ghana to 15% in Cameroon; however, the corresponding values for women and children ranged from 33%–38% in Ghana to 49%–73% in Cameroon (Figure 4).

In Cameroon, estimates derived from the 24hr and HCES data were qualitatively similar to those derived from household-level data. Estimated prevalence of high micronutrient intakes (intake or apparent nutrient density exceeding the UL) was low but not always zero (e.g., ∼5% for vitamin A among children in Cameroon, 1% for iron among men in Cameroon and Ghana, and 2%–9% for zinc among children in all surveys).

### Potential contribution of fortified bouillon to adequacy of (apparent) micronutrient intake

Fortified bouillon was predicted to reduce dietary vitamin A inadequacy substantially (Figure 1). For example, depending on the age and sex group (men, women, or preschool children), the addition of 120 μg/g bouillon was predicted to reduce inadequacy by ∼15–33 percentage points (pp) in Cameroon, ∼15–20 pp in Ghana, and ∼39–40 pp in Haiti (Figure 1). However, estimates of preformed retinol intake suggested that >5% of children might exceed the UL at fortification levels of 80 μg/g in Ghana, 120 μg/g in Cameroon, and 160 μg/g in Haiti.

For folate, at a fortification level of 80 μg folic acid/g, the estimated prevalence of folate inadequacy was reduced from 25%–51% to <15% in Ghana, from 20%–43% to <12% in Cameroon, and from 3%–8% to <1% in Haiti (Figure 2). At this level, median total folic acid intakes would be 206 (HCES) or 284 (24hr) μg folic acid/d among women in Cameroon or 246 μg/d in Ghana, which is still below the recommended additional 400 μg/d for women who may become pregnant [69]; in Haiti, the median total folic acid intakes (371 μg/d) would be similar to this threshold. However, the results suggested context-specific tradeoffs between meeting thresholds for adequacy and exceeding the UL (or corresponding nutrient density threshold): at 80 μg/g folic acid in bouillon, intakes were predicted to exceed the UL for 1% of children in Haiti, 4% of children in Cameroon, and 8% of children in Ghana.

For vitamin B12, bouillon fortification was predicted to have the largest impact on inadequacy in Haiti, where vitamin B12 inadequacy was reduced from 94%–98% to 4%–7% with the addition of vitamin B12 to bouillon cube at 2 μg/g (Figure 3). In all countries, vitamin B12 inadequacy was <20% for all target groups at fortification levels of 1.2 μg/g and above.

Bouillon fortification was predicted to have only a modest impact on the prevalence of dietary iron adequacy in the primary analysis (assuming 2% absorption of iron from bouillon) (Figure 4). At a level of 5 mg iron/g (∼8 times higher than bouillon currently fortified with iron on a voluntary basis [18]), the reduction in dietary inadequacy would be 3–17 pp across surveys and target groups (8–17 pp among women and children). At this same level of fortification, in scenarios assuming 4% and 10% absorption of iron in bouillon, the prevalence of dietary inadequacy could be reduced by 15–29 pp or 20–45 pp among women and children (Figure 5). At the same time, total iron intakes were predicted to exceed the UL for >5% of men at levels above 1.8 mg/g in Ghana and 5 mg/g in Haiti and Cameroon.

**FIGURE 6:**
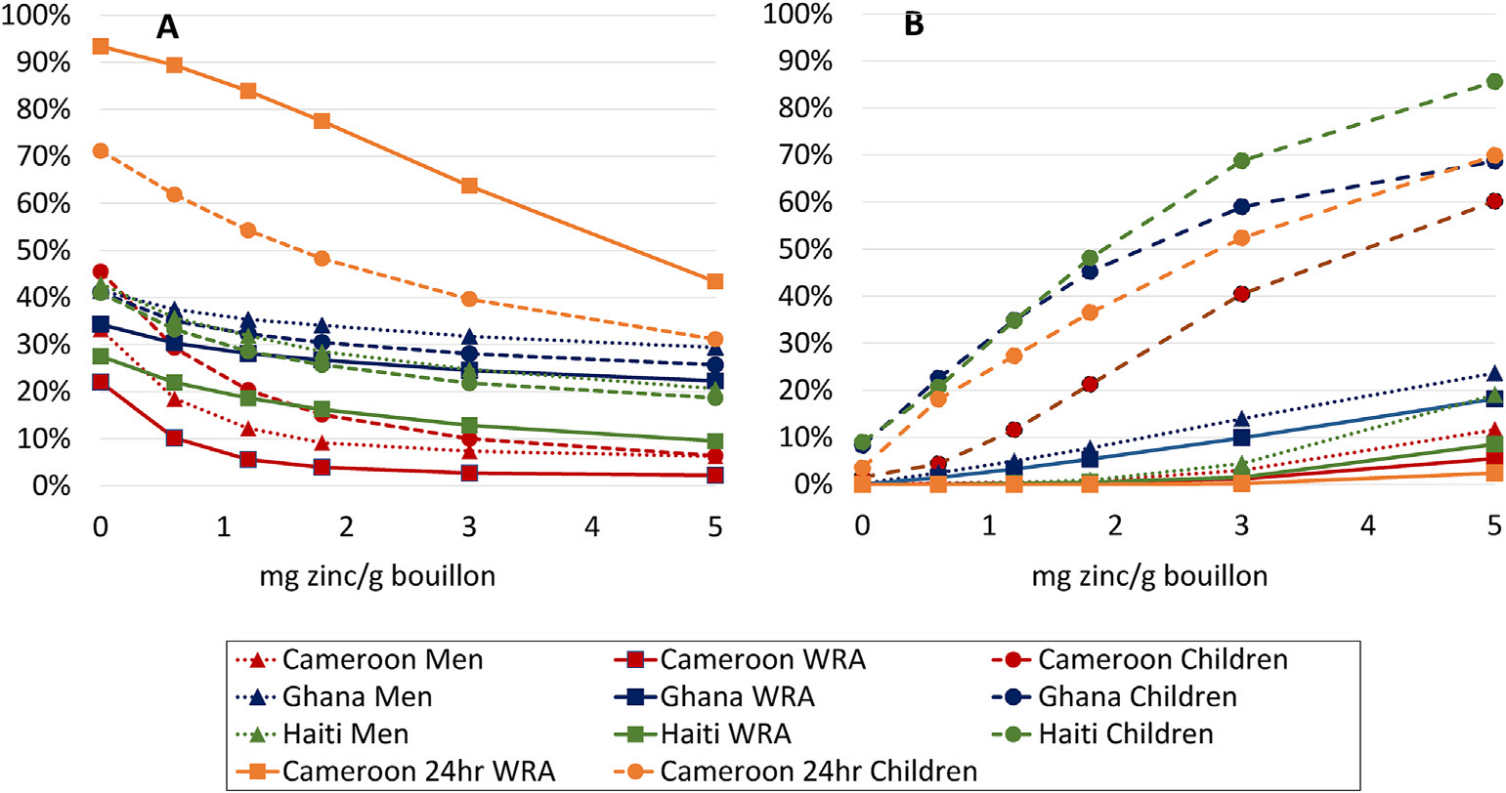
Prevalence of (A) inadequate and (B) high intake (Cameroon 24hr) or apparent nutrient densities (all others) of zinc among women of reproductive age, men, and 6- to 59-mo-old children at different assumed levels of zinc added to bouillon. Inadequate intake [apparent nutrient density] was defined as absorbable zinc intakes [zinc density of the household diet] below the age- and sex-specific estimated average requirement. Absorbable zinc was estimated using algorithms published by Miller and colleagues for women [54] and children [55]. High intake [apparent nutrient density] was defined as total zinc intake [zinc density of the household diet] above the tolerable upper intake level age- and sex-specific tolerable upper density]. Simulations account for fortification of wheat flour at estimated current levels (31.35 mg/kg in wheat flour for Cameroon; 22.8 mg/kg Haiti; 11.0 mg/kg Ghana). Technical considerations may limit the feasibility of certain fortification levels; concentrations ≤3 mg/g have been successfully added to bouillon in a research context [68].

With the addition of 3 mg/g zinc to bouillon, the modeling predicted a reduction of 9–13 pp among adults and children in Ghana, compared with reductions of 15–19 pp in Haiti and 19–36 pp in Cameroon (Figure 6). Among children, zinc intakes were predicted to exceed the UL (or corresponding density threshold) among 2%–9% of children in the absence of bouillon fortification, and among >40% of children in all countries at levels of 3 mg/g or greater. Among adults, high zinc intakes exceeded 5% at levels of 1.2 mg/g in Ghana and 5 mg/g in Cameroon and Haiti.

### Sensitivity analyses for ongoing mandatory fortification programs

Increasing the fortification levels in existing fortified products to the target levels (compared with estimates of the “status quo” scenario in the primary results reported above) resulted in lower prevalences of nutrient inadequacy, with the magnitude of difference depending on the country and nutrient (Supplemental Figures 1–5). For example, in Ghana, folate inadequacy among WRA was predicted to decrease from 51% to 26% if micronutrient concentrations in wheat flour were increased to match the target levels, but iron inadequacy would drop from 38% to 32% and B12 inadequacy would decrease only from 9% to 8%. Under these counterfactual scenarios, the predicted marginal impacts of bouillon fortification on dietary adequacy were generally lower, and the prevalence of intakes above the UL was greater, compared with those in the primary analyses, although in many cases the difference was <5 pp. Bouillon fortification was predicted to further reduce nutrient inadequacy particularly in cases in which the prevalence remained elevated even if existing programs were implemented according to standards (e.g., ≥18 percentage point reductions following bouillon fortification with vitamin A and folate in Ghana, with vitamin A and zinc in Cameroon, or with vitamin A in Haiti).

### Levels of fortification to meet dietary gaps

Based on the foregoing modeling results, we examined the range of fortification levels for bouillon to identify levels that would be consistent with WHO guidance to minimize inadequate intakes while avoiding intakes above the UL, using approaches based on (1) point estimates, or (2) lower 95% confidence intervals (Supplemental Table 8). Few scenarios examined met the strict criterion of no >0% intakes above the UL (point estimate or lower CI < 0.5%), except for vitamin A in Haiti (predicted to reduce dietary inadequacy by 18 pp), folic acid in Cameroon (predicted reduction of 10–28 pp) and Haiti (3–7pp), and iron in Haiti and Cameroon (both predicted reductions of ≤7 pp). With slightly more relaxed criteria for defining high intakes (point estimates <5% or <10%, or lower 95% CI <5%), acceptable fortification levels would range from 40 to 200 μg VA/g, 40 to 120 μg folic acid/g, or 0.6 to 3 mg iron/g, depending on the survey. However, only in the case of folate in Haiti was the predicted probability of inadequate intakes <2.5% for the population subgroups modeled.

As no UL for vitamin B12 has been defined and vitamin B12 is unlikely to have adverse organoleptic effects, the amount of vitamin B12 in bouillon could theoretically be increased until dietary adequacy is met for all regular bouillon consumers. At the fortification levels modeled, 2 μg/g would reduce inadequate apparent intake to ≤22% for all countries and target groups.

For zinc, none of the modeled levels of bouillon fortification met the criteria because zinc intake among children exceeded the selected thresholds at all levels of bouillon fortification (and in some cases in the absence of bouillon fortification). However, excluding children’s UL from consideration, levels of 0.6–3 mg/g would meet the selected criteria for adults, depending on the criterion and country.

## Discussion

Although some progress has been made in improving micronutrient status globally, further efforts are needed to reach individuals who remain at risk for deficiency; modeling of dietary data can help evaluate candidate strategies. In this analysis of national data from Cameroon, Ghana, and Haiti, we found that the prevalence of dietary micronutrient inadequacy varied across countries and target groups but exceeded 30% in many instances, even when accounting for the estimated contributions of current levels of fortification of staple foods, such as wheat flour and cooking oil. As expected, bouillon was commonly consumed, with 71% to >90% of households reporting bouillon consumption during the respective recall periods. Although the results varied by survey and target group, generally the modeling results suggest that bouillon could contribute to reducing dietary micronutrient inadequacy, with reductions of >20 pp in many cases.

A notable exception was iron, for which predicted estimates of change in dietary adequacy were modest (<17 pp reduction). The assumption of 2% absorption for the primary analysis was based on previous studies of the absorption of iron as ferric pyrophosphate (FePP) in bouillon [19,21]. However, new formulations may increase the absorption of iron [70], and absorption is likely to be greater among individuals with depleted iron stores [20,22], although further work would be needed to model the impact on absorption from the total diet. In sensitivity analyses, we found that increasing absorption of iron from bouillon to 4% or 10% would increase the predicted impact of dietary adequacy: at 10% absorption, reductions in dietary inadequacy of >20% could be achieved at 1.8 mg/g. Advantages of FePP, compared with other fortificants, include the lower likelihood of inducing organoleptic changes in the food [71,72]. Because absorption from FePP tends to be lower as well, the benefits of iron fortification with FePP are likely to be greatest in contexts with a high prevalence of iron deficiency and may be increased by innovations such as additives to enhance absorption [70]. Other strategies may be more effective or efficient for reducing dietary iron inadequacy (or preventing iron-deficiency anemia, a common policy goal); this question can be addressed by future modeling analyses.

The marginal contributions of bouillon fortification were lower under the assumption that foods included in ongoing mandatory fortification programs (e.g., wheat flour and cooking oil) were fortified at target levels. However, in many cases the estimates of reductions in dietary inadequacy attributable to fortified bouillon were >15 pp, suggesting that, even in the presence of well-performing LSFF programs, a fortified bouillon program may further contribute to achieving adequate micronutrient intakes. Compliance with fortification standards remains a major challenge for fortification programs globally [4], and the types and magnitudes of investments required to ensure that programs are implemented to standard may be substantial. In the absence of such investments, the “status quo” modeling scenarios based on observed micronutrient levels in products subject to mandatory fortification represent the current best estimate of the additional impact of introduction of fortified bouillon on dietary adequacy.

Although previous work suggested that bouillon fortification is less likely to lead to excessive intakes compared with food vehicles with more skewed intake distributions such as wheat flour [73], these modeling results suggest that, for some micronutrients, tradeoffs nevertheless exist between reducing dietary inadequacy and exceeding the UL for this delivery vehicle, too. WHO fortification guidelines rightly emphasize that joint consideration of both low and high intakes is necessary for planning fortification of condiments such as bouillon; our results illustrate the practical challenges in following WHO guidance of minimizing inadequacy while completely avoiding intakes above the UL when setting fortification levels. Two points should be borne in mind when considering the estimates of intake above the UL. First, the HCES has several limitations for estimating individual intake, as described below. One that is particularly relevant to estimating intakes above the UL is that estimates of apparent consumption are sometimes collected as household food acquisition or expenditures rather than asking directly about household consumption (i.e., use) during the observation period; this was the case for the household surveys conducted in Cameroon and Ghana. These values for food acquisition or expenditure are often observed to have extreme values at the upper tail of the distribution that may represent “real” episodic acquisition of foods but not actual consumption. We attempted to minimize the influence of these values by truncating values above 95th percentile (although there is no “gold standard” for this analytical choice) and by evaluating the adequacy and excessive intake using the nutrient density of the diet. Even when analyzing individual dietary intake data, it is difficult to precisely estimate the tails of the distributions, especially for nutrients such as retinol or folic acid that are often episodically consumed. These analytical limitations suggest that the interpretation of the prevalence of intakes above the UL should not be overly strict.

Second, the UL has a number of limitations as a reference value [73,74], including that the severity of the critical endpoints used to set each UL differs greatly by nutrient and many UL values (especially for children) are set based on limited or no data for that target group. Interpretation of the prevalence of intakes above the UL should therefore include an assessment of the health effects of exceeding the UL. For example, there is general consensus that zinc intakes above the UL for young children are not harmful, given the lack of direct data and the uncertainty around clinical effects of the critical endpoint of altered copper metabolism [67,73]. However, the consequences of high iron intakes for young children in contexts with frequent exposure to malaria and intestinal parasites likely merit more concern, even considering iron intake below the UL (which at 40 mg/d for children is quite high relative to the UL for adults of 45 mg/d) [75]. Policy decisions must therefore consider both the prevalence of low and high intakes as well as the potential severity of consequences of each [15].

Availability of appropriate data remains a major barrier to implementing the modeling approach recommended by WHO for setting fortification levels. Although HCES data have the advantages of being available for most countries, nationally representative, and having large sample sizes, the question of intrahousehold distribution remains a fundamental limitation for quantifying nutrient intakes for target groups of interest [29]. Other limitations of HCES for estimating dietary intake include incomplete food lists and inadequate accounting of food produced at home and/or food consumed away from home. The nutrient density method may help partially correct for misreporting of total household food consumption but requires further testing to understand performance of this approach in relation to estimation of the risk of nutrient intake below the EAR with individual intake data, and how this may vary by context. Despite these limitations, HCES analyses appear to generally yield results that are qualitatively similar to estimates from individual dietary surveys, suggesting they are useful for informing policy decisions [[27], [28], [32]]. Moreover, HCES data are preferable to the alternative of relying on Food Balance Sheets, which provide a national-level estimate of availability of specific commodities but (among other shortcomings) offer no information on overlap in consumption of different fortified foods.

Even when 24hr data are available from individuals, use of dietary intake data also has limitations with regard to assessing risk of deficiency. Particularly for minerals, numerous studies have observed inconsistency with patterns of risk as estimated from dietary intake compared with biomarkers (e.g., higher zinc intake in areas with lower serum zinc concentration [44]). These differences may be driven by factors that affect absorption and metabolism of iron and zinc, including the role of infections. For iron, although an absorption prediction equation that accounts for iron status (plasma ferritin concentrations) could be applied, we still had to rely on assumptions about the proportion of iron consumed as heme iron, which introduces uncertainty in the results. Improved modeling methods may help align these data in the future; however, it remains important to review biomarker data in addition to dietary intake data to assess deficiency risk and evaluate whether additional interventions to improve micronutrient status are warranted. Also, this analysis focused on the prevalence of nutrient inadequacy; however, improvements in dietary intake, even below the threshold for adequacy, may improve micronutrient status and contribute to mitigating the consequences of deficiency. In this study, patterns of dietary inadequacy were generally similar to those observed in biomarker surveys in Cameroon [14] and Ghana [63] in terms of the public health relevance of deficiencies in these micronutrients, although some exceptions were observed (e.g., iron and zinc in Cameroon). Estimation of bouillon intake is also subject to numerous sources of error, and we are not aware of studies that examine the validity of different methods for assessing bouillon intake. Despite these limitations for setting fortification levels with precision, the results represent an initial step in assessing the potential impacts of bouillon fortification and identifying a range of fortification levels to balance inadequate and high micronutrient intakes.

Following WHO guidance, we identified fortification levels, which would increase dietary adequacy while maintaining the prevalence of high intakes (i.e., those above the UL) below selected thresholds. These values should be interpreted with caution given the aforementioned limitations of HCES for establishing bouillon fortification standards. In addition, although we accounted for the current performance of existing mandatory LSFF programs, the data sources for Cameroon and Haiti are each over 10 y old and so these results may not reflect current dietary intakes, which could be expected to improve in nutrient adequacy over time [76]. In Haiti, diets may be different because the 2012 ECVMAS was performed 2 y after a major earthquake that caused internal displacement, disruption of market supply, and changes in the price of staple foods. However, the values represent a starting point for country-level discussions on bouillon fortification standards. We limited analyses to examination of multiple physiological groups at the national level, consistent with the level of implementation of LSFF programs. Assessment of how population subgroups, such as geographic region or wealth quintile, might be impacted may further inform country-level policy discussions. Countries considering bouillon fortification might also make use of modeling scenarios to examine the potential impacts of assumptions around compliance with fortification policy and nutrient retention. Likewise, examining the overlap with other intervention programs, such as distribution of micronutrient powders or vitamin A supplements, could help inform development of a coherent micronutrient strategy to balance risk of low and high intakes.

Decisions about fortification policy will also require inputs beyond the predicted nutrition and public health impacts. For example, because of the low intake of bouillon compared with other fortified staple foods such as cereal flours, technical challenges may arise in adding a very concentrated micronutrient premix to the cube. Thus, there is a need to evaluate the technical feasibility of these levels and any impacts on sensory properties, especially for iron. Recent work confirmed the acceptability of a multiple micronutrient-fortified bouillon cube (containing 200 μg VA/g, 80 μg folic acid/g, 1.2 μg B12/g, 4 mg iron/g, and 3 mg zinc/g) among women in northern Ghana [68]; however further efforts are needed to understand what amounts are feasible under commercial storage and transport conditions. These discussions must necessarily take into account the cost of fortification and which stakeholder groups will be called upon to pay these costs, the profitability and consumer affordability of the final retail product, and the feasibility and affordability of regulatory and other government investments and activities associated with voluntary or mandatory standards. These challenges may affect the types and amounts of micronutrients that are technically and otherwise feasible to add to a multiple micronutrient-fortified bouillon cube.

As hypertension is increasingly recognized as a public health priority in low- and middle-income countries [15], discussions on bouillon fortification have also included concerns about the sodium content of this potential vehicle, which is ∼20%–30% sodium by weight [13,16]. Some studies have suggested that bouillon contributes only a modest proportion of total salt consumption (∼10%–25%), whereas other studies suggest that bouillon made with iodized salt can contribute substantially to iodine intake [30,31,77]. The dual public health objectives of sodium reduction and micronutrient fortification of salt and bouillon need not be mutually exclusive [13], and data on micronutrient intakes as well as sources of dietary salt or sodium can inform both strategies.

In conclusion, in countries where bouillon is a commonly consumed condiment, a few fortification vehicles, perhaps other than iodized salt, reach a similarly high proportion of people, including people in rural areas and in households with limited resources. These modeling results from Cameroon, Ghana, and Haiti indicate that fortification of bouillon has the potential to reduce dietary nutrient inadequacy, even in the presence of current programs to fortify other staple foods. Alternative strategies may be needed for iron, for which bouillon fortification is predicted to have a smaller impact on dietary adequacy unless absorption can be enhanced. Decisions must consider carefully the health and other consequences of deficiency as well as those associated with high intakes of each micronutrient. Further work is needed to identify fortification levels that will meet criteria for nutritional benefits, technical and commercial feasibility, cost-effectiveness, and affordability.

## Author contributions

The authors’ responsibilities were as follows – RES, SMK, HL, SAV, KPA: designed the research; SMK, HL, EG, KPA: analyzed data; RES: wrote the paper and had primary responsibility for final content; and all authors contributed to data interpretation and critically reviewed the manuscript and read and approved the final manuscript.

## Funding

This analysis was supported, in part, by a grant from Helen Keller International (66504-UCD-01) to the University of California, Davis, through support from the Bill & Melinda Gates Foundation (INV-007916). Under the grant conditions of the Foundation, a Creative Commons Attribution 4.0 Generic License has already been assigned to the Author Accepted Manuscript version that might arise from this submission. Collection of dietary intake data in Cameroon was supported by the Michael and Susan Dell Foundation.

## Data availability

The national survey data analyzed for this manuscript may be requested from the cited institutions.

## Conflict of interest

Reina Engle-Stone is an Editorial Board Member for Current Developments in Nutrition and played no role in the Journal’s evaluation of the manuscript. All other authors report no conflicts of interest.
