# Supplementary material for: The Potential for Bouillon Fortification to Reduce Dietary Micronutrient Inadequacy: Modeling Analyses Using National Survey Data from Cameroon, Ghana, and Haiti
Source: Curr Dev Nutr. 2024 Oct 18;8(11):104485. doi: 10.1016/j.cdnut.2024.104485 (PMC11617227; doi:10.1016/j.cdnut.2024.104485)
Supplement: multimedia component 1 [file mmc1.docx]

**Supplemental Material**

**Supplemental Table 1: Summary of methods, definitions and thresholds for primary analysis of each nutrient**^1^

| Nutrient | Outcome | Definition (24hr) | Definition (HCES) |
| --- | --- | --- | --- |
| Vitamin A | Inadequacy | Total vitamin A intake (µg RAE/d) < EAR | Vitamin A density of the household diet < CND |
|  | Excess | Retinol intake (µg/d) > UL | Retinol density of the household diet > UD |
| Folate | Inadequacy | Total folate intake (µg DFE/d) < EAR | Folate density of the household diet < CND |
|  | Excess | Folic acid intake (µg/d) > UL | Folic acid density of the household diet > UD |
| Vitamin B12 | Inadequacy | Absorbable vitamin B12^2^ (µg/d) < modified EAR | Total vitamin B12 density of the household diet < CND |
| Iron | Inadequacy | Intake of absorbable iron (mg/d) calculated by the full probability method. Absorbable iron calculated by assuming 10% heme iron and 25% absorption of heme iron, and applying algorithm by Armah et al. based on ferritin and phytate only. Iron from breast milk was assumed to be 0.0549 mg/d (based on 0.2 mg iron/L, 0.549 L milk/day, and 50% absorption of iron from human milk([1](#_ENREF_1))). Absorption from iron-fortified bouillon was assumed to be 2% in the primary analysis. | For non-pregnant women and for children, calculated using the full probably method based on the iron density of the household diet. For pregnant women and men: Total iron density of the household diet < CND adjusted for assumed absorption. Absorption assumed to be 10% from background diet and 2% from iron-fortified bouillon. |
|  | Excess | Total iron intake (mg/d) > UL | Total iron density of the household diet > UD |
| Zinc | Inadequacy | Absorbable zinc intake (mg/d) < EAR.  Absorption estimated using algorithms for adults ([2](#_ENREF_2)) and children ([3](#_ENREF_3)) | Absorbable zinc density of the household diet < adjusted CND^3^ |
|  | Excess | Total zinc intake (mg/d) > UL | Total zinc density of the household diet > UD |

^1^CND, critical nutrient density; EAR, estimated average requirement; HCES, household income and expenditure survey; UD, tolerable upper density; UL, tolerable upper intake level

^2^Absorption estimated using a modified version of the algorithm published by Doets et al., consistent with previous analyses ([4](#_ENREF_4), [5](#_ENREF_5)). In a sensitivity analysis, the estimated prevalence of dietary B12 inadequacy among children was 36.2% based on absorbable B12 and 29.0% based on total B12 and the impact of B12 fortification of bouillon was similar between the two methods (~7 percentage point reduction in inadequacy).

^3^Adjusted critical nutrient densities were calculated based on survey-specific median percent absorbed zinc among children and, separately, women of reproductive age.

**Supplemental Table 2: Sources of estimates of breastmilk nutrient intake for vitamin A, folate, vitamin B-12, iron, and zinc for breastfed children used for analysis of Cameroon National Micronutrient Survey data**

| Nutrient | Macro-region | Breastmilk nutrient concentration | Breastmilk nutrient concentration | Average breastmilk intake^6^ | Breastmilk nutrient intake |
| --- | --- | --- | --- | --- | --- |
| Vitamin A^1^ | South | 30.4 µg/g fat | 1.003 µg/g milk | 549 g/d | 551 µg/d |
|  | North | 12.8 µg/g fat | 0.423 µg/g milk^1^ | 549 g/d | 223 µg/d |
|  | Yaoundé/Douala | 26.1 µg/g fat | 0.862 µg/g milk^1^ | 549 g/d | 473 µg/d |
| Folate^2^ | South, North, Yaoundé/Douala | - | - | - | 49 µg/d |
| Vitamin B-12^3^ | South | 236 pmol/L | 319.78 pg/ml | 549 g/d | 0.170 µg/d |
|  | North | 47 pmol/L | 63.69 pg/ml | 549 g/d | 0.034 µg/d |
|  | Yaoundé/Douala | 286 pmol/L | 387.53 pg/ml | 549 g/d | 0.207 µg/d |
| Iron^4^ | South, North, Yaoundé/Douala | - | - | - | 0.0549 mg/d |
| Zinc^5^ | South, North, Yaoundé/Douala | - | - | - | 0.145 mg/d |

^1^Assuming 34 g fat/L and specific gravity of milk = 1.03 g/mL; Breastmilk vitamin A concentration (µg/g milk) = Breastmilk vitamin A concentration (µg/g fat) * 34 g fat/L / (1.03 g/mL * 1000); Breastmilk vitamin A intake = Breastmilk vitamin A concentration (µg/g) * average breastmilk intake (g/d).

^2^Folate: Assumed an average contribution of 49 µg/d based on Brown et al., 1998 ([6](#_ENREF_6))

^3^B-12 concentration: 1 pmol/l = 1.355014 pg/ml. Breastmilk B-12 concentration (pg/ml) = breastmilk B-12 concentration (pmol/L) * 1.355014; Breastmilk B-12 intake = breastmilk B-12 concentration (pg/ml) * average breastmilk intake (g/d)/(1.03 g/ml * 10^6^).

^4^Value calculated based on the following assumptions: 0.2 mg iron/L, 0.549 L milk/day, and 50% absorption of iron from human milk ([1](#_ENREF_1)).

^5^ Based on Brown *et al*’s estimate([7](#_ENREF_7)), the zinc intake from breastmilk for partially breastfed infants age 12-17 months estimated was 0.29 mg/day. We assumed that the bioavailability of zinc from breast milk is 50%, therefore, zinc intake from breastmilk is 0.145 mg/d.

^6^ Estimated average daily milk intake for partially breastfed children 12-23 mo of age in developing countries ([6](#_ENREF_6))

**Supplemental Table 3: Estimated Average Requirements, Critical Nutrient Densities, and Energy Requirements**^1^

| Target group | Age and physiological status | Vitamin A (µg RAE) | | Folate (µg DFE) | | Total B12 (absorbed B12^2^), µg/d | | Absorbed Zinc (mg/d) | | Energy (kcal/d)^3^ |
| --- | --- | --- | --- | --- | --- | --- | --- | --- | --- | --- |
|  |  | EAR | CND | EAR | CND | EAR | CND | EAR | CND |  |
| Men | 18 y | 630 | 217 | 330 | 114 | 2.0 | 0.69 | 3.7 | 1.28 | 2,900 |
|  | 19-50 y | 625 | 216 - 222 | 320 | 110 - 114 | 2.0 | 0.69 - 0.71 | 3.7 | 1.28 - 1.32 | 2,813-2,900 |
|  | 51-60 y | 625 | 222 | 320 | 114 | 2.0 | 0.71 | 3.7 | 1.32 | 2,813 |
| Women | NPNL, 14-18 | 485 | 204 - 212 | 330 | 139 - 144 | 2.0 (1.0) | 0.84 - 0.87 | 2.89 | 1.22 - 1.26 | 2291-2378 |
|  | NPNL, 19-50 y^4^ | 500 | 218 - 224 | 320 | 140 - 143 | 2.0 (1.0) | 0.87 - 0.90 | 2.89 | 1.26 - 1.29 | 2233-2291 |
|  | Pregnancy, 14-18 y | 530 | 190 - 196 | 520 | 186 - 192 | 2.2 (1.1) | 0.79 - 0.81 | 3.59 | 1.28 - 1.33 | 2709-2796 |
|  | Pregnancy, 19-50 y | 550 | 203 - 207 | 520 | 192 - 196 | 2.2 (1.1) | 0.81 - 0.83 | 3.59 | 1.33 - 1.35 | 2651-2709 |
|  | Lactation, 14-18 y | 885 |  | 450 |  | 2.4 (1.2) |  | 3.89 |  | n/a |
|  | Lactation, 19-50 y | 900 |  | 450 |  | 2.4 (1.2) |  | 3.89 |  | n/a |
| Children | ≤ 3 y | 210 | 196 - 329 | 120 | 112 - 188 | 0.7 (0.35) | 0.65 - 1.10 | 1.074 | 1.00 - 1.68 | 638-1073 |
|  | 4-5 y | 275 | 216 - 250 | 160 | 125 - 145 | 1.0 (0.5) | 0.78 - 0.91 | 1.390 | 1.09 - 1.26 | 1102-1276 |

^1^EAR, estimated average requirement; CND, critical nutrient density DFE, dietary folate equivalents; NPNL, non-pregnant, non-lactating women; RAE, retinol activity equivalents. All micronutrient reference values were taken from the IOM Dietary Reference Intakes ([8](#_ENREF_8)), except for zinc: for women, the corrected IZiNCG values were used ([9](#_ENREF_9)) and for children, the EFSA physiological requirements were used ([10](#_ENREF_10)). For iron, the full probability method was used to estimate inadequate absorbable iron intake (or absorbable iron density, in the HCES data) using tables from IOM ([8](#_ENREF_8)).

^2^The EAR for total B12 was applied for analysis of HCES data, while the EAR for absorbable B12 (IOM assumed 50% absorption of dietary B12) was applied for the 24-hr data, as described in Supplemental Table 1.

^3^Energy requirements were estimated relative to the FAO human energy requirement estimate of 2,900 kcals for an 65kg adult male age 18-30 years with moderate physical activity (FAO)([11](#_ENREF_11)). Additional energy requirements in pregnancy were estimated as the average of 360 additional kcal/d during the second trimester and 475 kcal/d during the third trimester (average of 417.5 kcal/d) (FAO). Age- and sex-specific critical nutrient densities were calculated as the EAR divided by energy requirements, multiplied by 1,000 kcals.

^4^In the 24-h recall data, for women with missing age, we assumed their age to be 19-50 y. For children with missing age, we assumed age to be ≤ 3 y.

**Supplemental Table 4: Tolerable Upper Intake Levels**^1^

| Target group | Age and physiological status | Retinol^2^, µg/d | Folic acid^3^, µg/d | Total iron, mg/d | Total Zinc, mg/d | Energy (kcal/d)^4^ |
| --- | --- | --- | --- | --- | --- | --- |
| Men | 18 y | 2,800 | 800 | 45 | 40 | 2,900 |
|  | 19-50 y | 3,000 | 1,000 | 45 | 40 | 2,813-2,900 |
|  | >50 y | 3,000 | 1,000 | 45 | 40 | 2,813 |
| Women | NPNL, 14-18 | 2,800 | 800 | 45 | 40 | 2291-2378 |
|  | NPNL, 19 – 50 y^5^ | 3,000 | 1,000 | 45 | 40 | 2233-2291 |
|  | Pregnancy, 14 – 18 y | 2,800 | 800 | 45 | 40 | 2709-2796 |
|  | Pregnancy, 19 – 50 y | 3,000 | 1,000 | 45 | 40 | 2651-2709 |
|  | Lactation, 14-18 y | 2,800 | 800 | 45 | 40 | n/a |
|  | Lactation, 19 – 50 y | 3,000 | 1,000 | 45 | 40 | n/a |
| Children | ≤ 3 y | 600 | 300 | 40^2^ | 7 | 638-1073 |
|  | 4-5 y | 900 | 400 | 40 | 12 | 1102-1276 |

^1^NPNL, non-pregnant, non-lactating women. B12 is not included because no UL has been defined for B12. All values from the IOM Dietary Reference Intakes ([8](#_ENREF_8)).

^2^The UL for vitamin A applies to preformed vitamin A only, including vitamin A from animal sources, fortified food, and supplements.

^3^The Ul for folate applies to synthetic forms (i.e., folic acid) obtained from supplements, fortified foods, or a combination of two.

^4^Energy requirements were estimated relative to the FAO human energy requirement estimate of 2,900 kcals for an 65kg adult male age 18-30 years with moderate physical activity (FAO)([11](#_ENREF_11)). Additional energy requirements in pregnancy were estimated as the average of 360 additional kcal/d during the second trimester and 475 kcal/d during the third trimester (average of 417.5 kcal/d) (FAO). Age- and sex-specific tolerable upper densities were calculated as the UL divided by energy requirements, multiplied by 1,000 kcals.

^5^In the 24-h recall data, for women with missing age, we assume their age is 19-50 y. For children with missing age, we assume age is ≤ 3 y.

**Supplemental Table 5. Bouillon fortification levels tested^1^**

| **Micronutrient, units** | **Concentrations per gram bouillon** |
| --- | --- |
| Vitamin A, µg | 0, 40, 80, 120, 160, 200, 250 |
| Folic acid, µg | 0, 20, 40, 60, 80, 100, 120 |
| Vitamin B12, µg | 0, 0.2, 0.4, 0.8, 1.2, 1.6, 2.0 |
| Iron^2^, mg | 0, 0.6, 1.2, 1.8, 3.0, 5.0 |
| Zinc^2^, mg | 0, 0.6, 1.2, 1.8, 3.0, 5.0 |

^1^A large range of concentrations was selected for the purpose of this modeling exercise; technical feasibility of this entire range has not been confirmed, particularly for iron and zinc. However, a bouillon cube formulation for research has been developed containing 200 µg vitamin A, 80 µg folic acid, 1.2 µg vitamin B12, 4 mg iron, and 3 mg zinc per gram of bouillon: https://www.clinicaltrials.gov/study/NCT05178407

^2^Scenarios in which 8.0 mg iron or zinc per gram bouillon were modeled but were later dropped due to unlikely technical feasibility.

**Supplemental Table 6. Median (IQR) nutrient intake among women 15-49 y and children 6-59 mo in Cameroon, with mandated large-scale fortification programs at current operating levels**^1^

|  | Women 15-49 y | Children 6-59 mo |
| --- | --- | --- |
| Vitamin A, µg RAE/d | 648 (393, 857) | 283 (170, 430) |
| Retinol, µg/d | 98 (31, 257) | 69 (15, 238) |
| Folate, µg DFE/d | 393 (298, 502) | 200 (136, 281) |
| Folic acid, µg/d | 11 (1, 113) | --^2^ |
| Vitamin B12, µg/d | 1.3 (0.6, 2.5) | 0.6 (0.2, 1.3) |
| Iron, mg/d | 11.8 (8.9, 15.4) | 5.7 (4.0, 7.6) |
| Absorbable iron, mg/d | 1.0 (0.6, 1.5) | 0.6 (0.4, 0.9) |
| Zinc, mg/d | 9.0 (6.6, 12.2) | 4.9 (3.7, 6.1) |
| Absorbable zinc, mg/d | 1.6 (1.2, 2.2) | 0.98 (0.95, 1.01) |

^1^ Assumes that staple foods that are subject to mandatory micronutrient fortification are fortified at levels observed in recent program monitoring data, as described in the text. DFE, dietary folate equivalents; RAE, retinol activity equivalents.

^2^ Model did not converge

**Supplemental Table 7. Median (IQR) apparent household nutrient densities calculated from household surveys in Cameroon, Ghana, and Haiti, with mandated large-scale fortification programs at current operating levels**^1^

|  | Cameroon | Ghana | Haiti |
| --- | --- | --- | --- |
| Vitamin A density, µg RAE per 1,000 kcals | 290.6 (168.7, 431.6) | 280.0 (136.5, 626.0) | 186.2 (130.0, 253.2) |
| Retinol density, µg per 1,000 kcals | 38.5 (9.6, 122.5) | 68.7 (32.5, 122.3) | 129.5 (89.7, 180.7) |
| Folate, µg DFE per 1,000 kcals | 180.4 (130.2, 234.2) | 146.3 (109.7, 187.5) | 260.6 (200.1, 320.6) |
| Folic acid, µg per 1,000 kcals | 14.8 (0, 42.6) | 11.1 (2.8, 21.8) | 73.2 (45.8, 101.5) |
| Vitamin B12, µg per 1,000 kcals | 1.3 (0.6, 2.2) | 2.0 (1.1, 3.5) | 0.2 (0.1, 0.4) |
| Iron, mg per 1,000 kcals | 4.9 (3.9, 6.4) | 7.6 (6.4, 8.8) | 5.3 (4.3, 6.2) |
| Absorbable iron, mg per 1,000 kcals | 0.5 (0.4, 0.6) | 0.8 (0.6, 0.9) | 0.5 (0.4, 0.6) |
| Zinc, mg per 1,000 kcals | 4.3 (3.6, 4.9) | 5.1 (4.2, 5.9) | 4.9 (4.1, 5.7) |
| Absorbable zinc^2^, mg per 1,000 kcals, children | 1.1 (0.9, 1.2) | 1.4 (1.1, 1.6) | 1.3 (1.1, 1.5) |
| Absorbable zinc^3^, mg per 1,000 kcals, WRA | 1.6 (1.3, 1.8) | 1.7 (1.4, 2.0) | 1.8 (1.5, 2.1) |

^1^Assumes that staple foods that are subject to mandatory micronutrient fortification are fortified at levels observed in recent program monitoring data, as described in the text.

^2^This is based on median absorbed zinc of 25% for children in Cameroon, 27% in Ghana and 27% in Haiti. In practice, we applied an adjusted physiological requirement rather than an “absorbed zinc” nutrient density.

^3^This is based on median absorbed zinc of 37% for WRA in Cameroon, 34% in Ghana and 36% in Haiti. In practice, we applied an adjusted physiological requirement rather than an “absorbed zinc” nutrient density.

**Supplemental Table 8: Maximum fortification level associated with each threshold for acceptable prevalence of apparent intakes above the UL, and corresponding estimate of reduction in dietary inadequacy^1^**

|  |  | Point estimates | | | | | | | | Confidence intervals | | | | | |
| --- | --- | --- | --- | --- | --- | --- | --- | --- | --- | --- | --- | --- | --- | --- | --- |
|  |  | 0% exceeding UL for any target group | | < 5% exceeding UL for any target group | | <10% exceeding UL for any target group | | | 0% exceeding UL for any target group, based on lower 95% CI | | | | <5% exceeding UL for any target group, based on lower 95% CI | |  |
|  |  | Quantity per g bouillon | Reduction in inadequacy^2^, pp | Quantity per g bouillon | Reduction in inadequacy^2^, pp | Quantity per g bouillon | Reduction in inadequacy^2^, pp | Quantity per g bouillon | | | Reduction in inadequacy^2^, pp | Quantity per g bouillon | | Reduction in inadequacy^2^, pp |  |
| Vitamin A, µg | Cameroon | 0 | 0 | 80 | 12-26 | 200 | 19-37 | 0 | | | 0 | 200 | | 19-37 |  |
|  | Ghana | 0 | 0 | 40 | 8-11 | 80 | 13-17 | 0 | | | 0 | 40 | | 8-11 |  |
|  | Haiti | 40 | 18 | 120 | 39-40 | 160 | 45 | 40 | | | 18 | 120 | | 39-40 |  |
| Folic acid, µg | Cameroon | 0 | 0 | 80 | 11-31 | 100 | 12-34 | 60 | | | 10-28 | 100 | | 12-34 |  |
|  | Ghana | 0 | 0 | 40 | 15-32 | 80 | 16-36 | 0 | | | 0 | 60 | | 16-35 |  |
|  | Haiti | 60 | 3-7 | 120 | 3-7 | 120 | 3-7 | 80 | | | 3-7 | 120 | | 3-7 |  |
| Iron, mg | Cameroon | 0 | 0 | 3 | 5-9 | 3 | 8-11 | 0.6 | | | 1-2 | 3 | | 5-9 |  |
|  | Ghana | 0 | 0 | 0.6 | 1-3 | 1.8 | 2-7 | 0 | | | 0 | 0.6 | | 1-3 |  |
|  | Haiti | 1.2 | 3-4 | 3 | 5-11 | 3 | 5-11 | 1.8 | | | 4-7 | 3 | | 5-11 |  |
| Zinc^3^, mg | Cameroon | 0.6 | 4-16 | 3 | 19-36 | 3 | 19-36 | 1.8 | | | 16-30 | 3 | | 19-36 |  |
|  | Ghana | 0 | 0 | 0.6 | 4-6 | 1.8 | 7-11 | 0 | | | 0 | 1.2 | | 6-9 |  |
|  | Haiti | 1.2 | 9-12 | 3 | 15-19 | 3 | 15-19 | 1.2 | | | 9-12 | 3 | | 15-19 |  |

^1^ Target groups analyzed include men, women, and preschool children. Vitamin B12 is not included because no UL has been established for vitamin B12. For Cameroon, maximum fortification level was set based on the dataset with higher prevalence of intake above the UL (more conservative); range of decrease in dietary adequacy represents data from both surveys.

^2^Indicates reduction in prevalence of dietary micronutrient inadequacy, in percentage points (pp).

^3^Results represent the program planning scenario in which intake above the UL among preschool children is not considered. Total zinc intakes exceeded the specified thresholds for intakes > UL for preschool children in all datasets and therefore under these criteria no zinc would be added to bouillon. See manuscript text for discussion of interpretation of the UL for preschool children.

**
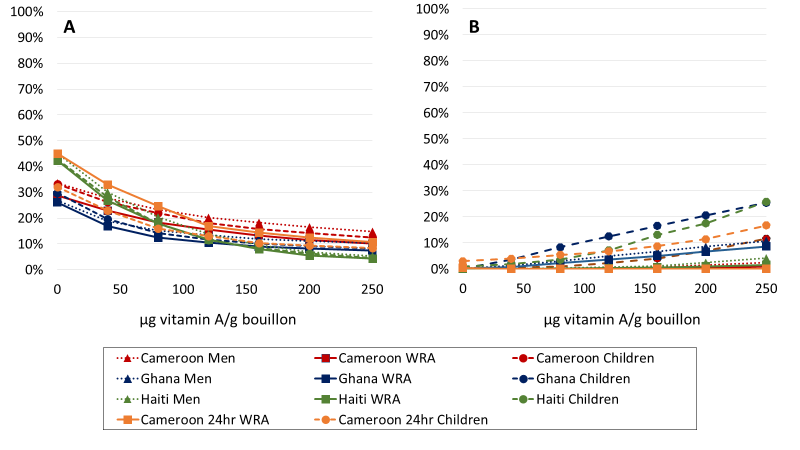
**

**Supplemental Figure 1.** Prevalence of (A) inadequate or (B) high intake (Cameroon 24-hour dietary recall) or apparent nutrient densities (all other datasets) of vitamin A among women of reproductive age, men, and children 6-59 months of age at different modeled levels of vitamin A added to bouillon. For 24-hour dietary recall data, inadequate intake was defined as total vitamin A (expressed as ug RAE/d) below the age- and sex-specific estimated average requirement while high intake was defined as preformed retinol (expressed as µg/d) above the tolerable upper intake level. For household consumption and expenditure survey data, inadequate apparent nutrient density was defined as vitamin A density of the household diet below the age- and sex- specific critical nutrient density, and high apparent nutrient density was defined as retinol density of the household diet above the age- and sex- specific tolerable upper density. Simulations account for fortification of cooking oil (12 mg/kg in oil for Cameroon; 15 mg/kg Haiti; 10 mg/kg Ghana) and wheat flour (2.0 mg/kg for Ghana) at the target concentrations specified in current fortification standards. Technical considerations may limit the feasibility of certain fortification levels; concentrations up to 200 µg/g have been successfully added to bouillon in a research context ([12](#_ENREF_12)). The prevalence of high retinol intakes among children in the absence of bouillon fortification was estimated without including the contribution of human milk due to challenges incorporating this information into the two-part version of the NCI model for estimating usual intake distributions.


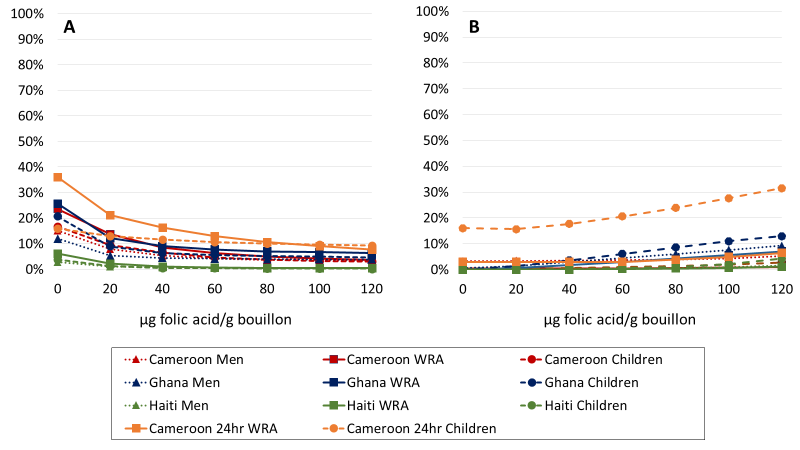


**Supplemental Figure 2.** Prevalence of (A) inadequate and (B) high intake (Cameroon 24hr) or apparent nutrient densities (all others) of folate among women of reproductive age, men, and children 6-59 months of age at different modeled levels of folic acid added to bouillon. For 24-hour dietary recall data, inadequate intake was defined as total folate (expressed as ug dietary folate equivalents [DFE]/d) below the age- and sex-specific estimated average requirement while high intake was defined as folic acid (expressed as µg/d) above the tolerable upper intake level. For household consumption and expenditure survey data, inadequate apparent nutrient density was defined as folate density of the household diet below the age- and sex- specific critical nutrient density, and high apparent nutrient density was defined as folic acid density of the household diet above the age- and sex- specific tolerable upper density. Simulations account for fortification of wheat flour at the target concentrations specified in current fortification standards (5 mg/kg in wheat flour for Cameroon; 1.5 mg/kg Haiti; 2.08 mg/kg Ghana). Technical considerations may limit the feasibility of certain fortification levels; concentrations up to 80 µg/g have been successfully added to bouillon in a research context ([12](#_ENREF_12)). Due to challenges implementing the two-part NCI model, the estimates of high folic acid intake in the absence of fortified bouillon could not be reliably estimated; therefore, we imputed the value for the lowest level of folic acid addition to fortified bouillon (estimated with the one-part NCI model).

**
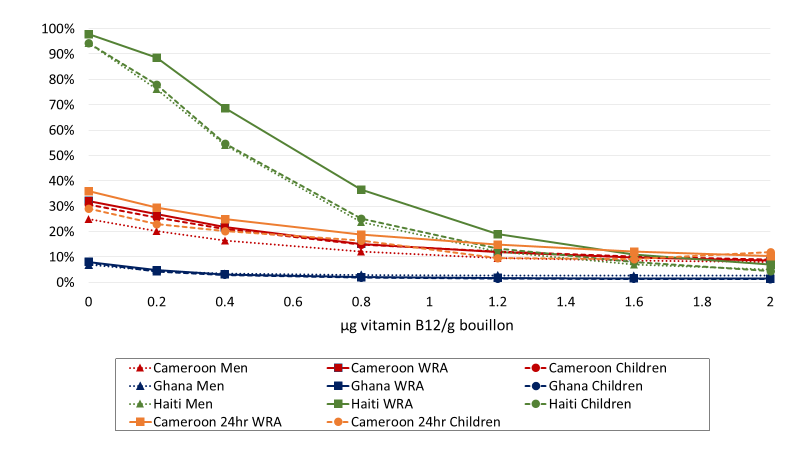
**

**Supplemental Figure 3.** Prevalence of inadequate intake (Cameroon 24hr) or apparent nutrient densities (all others) of vitamin B12 among women of reproductive age, men, and children 6-59 months of age at different modeled levels of vitamin B12 added to bouillon. For 24-hour dietary recall data, inadequate intake was defined as vitamin B12 (expressed as ug/d) below the age- and sex-specific estimated average requirement. For household consumption and expenditure survey data, inadequate apparent nutrient density was defined as vitamin B12 density of the household diet below the age- and sex- specific critical nutrient density. High intakes were not modeled because no tolerable upper intake level has been defined for vitamin B12. Simulations account for fortification of wheat flour at the target concentrations specified in current fortification standards (0.04 mg/kg in wheat flour for Cameroon; n/a Haiti; 0.01 mg/kg Ghana). Technical considerations may limit the feasibility of certain fortification levels; concentrations up to 1.2 µg/g have been successfully added to bouillon in a research context ([12](#_ENREF_12)).

**
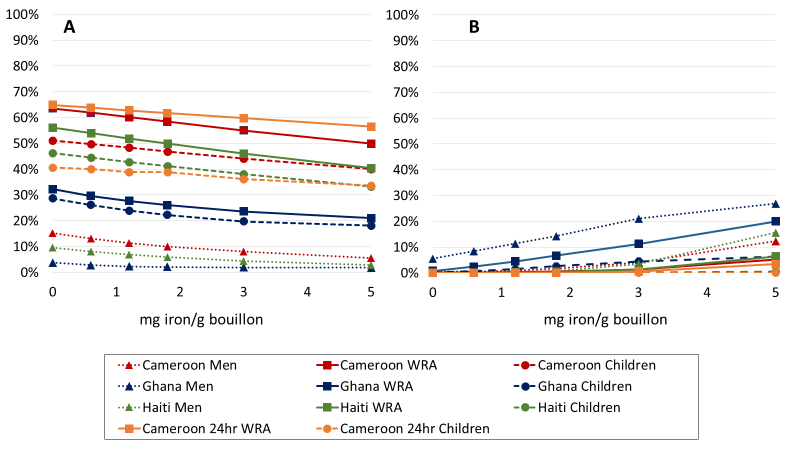
**

**Supplemental Figure 4.** Prevalence of (A) inadequate and (B) high intake (Cameroon 24hr) or apparent nutrient densities (all others) of iron among women of reproductive age, men, and children 6-59 months of age at different modeled levels of iron added to bouillon. Inadequate intake was calculated using the full probability approach as inadequate absorbable iron intake (24-hour dietary recall data) or iron density of the household diet (household consumption and expenditures surveys). Non-heme iron absorption was estimated according to Armah et al ([13](#_ENREF_13)) for the Cameroon 24-hour recall dataset (see Methods for details); in all other surveys, iron absorption was assumed to be 10% from the total diet. Iron absorption from fortified bouillon was assumed to be 2%. Simulations account for fortification of wheat flour at the target concentrations specified in current fortification standards (60 mg/kg in wheat flour for Cameroon; 30 mg/kg Haiti; 58.5 mg/kg Ghana). Technical considerations may limit the feasibility of certain fortification levels; concentrations up to 4 mg/g have been successfully added to bouillon in a research context ([12](#_ENREF_12)).


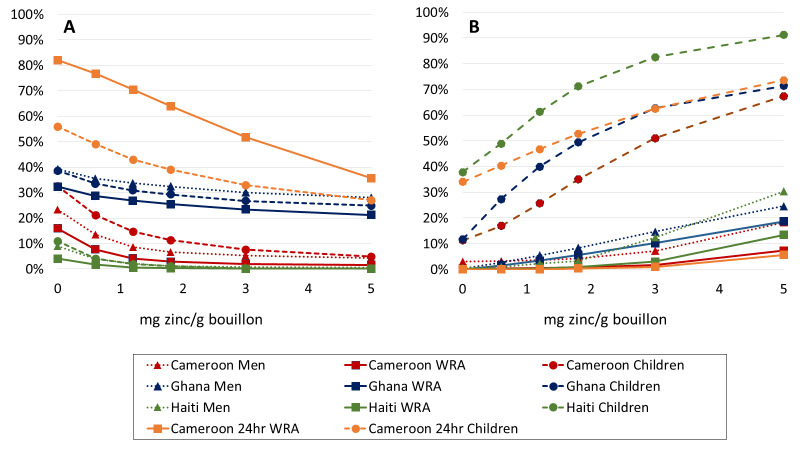


**Supplemental Figure 5.** Prevalence of (A) inadequate and (B) high intake (Cameroon 24hr) or apparent nutrient densities (all others) of zinc among women of reproductive age, men, and children 6-59 months of age at different assumed levels of zinc added to bouillon. Inadequate intake [apparent nutrient density] was defined as absorbable zinc intakes [zinc density of the household diet] below the age- and sex-specific estimated average requirement. Absorbable zinc was estimated using algorithms published by Miller and colleagues for women ([2](#_ENREF_2)) and children ([3](#_ENREF_3)). High intake [apparent nutrient density] was defined as total zinc intake [zinc density of the household diet] above the tolerable upper intake level age- and sex- specific tolerable upper density]. Simulations account for fortification of wheat flour at the target concentrations specified in current fortification standards (95 mg/kg in wheat flour for Cameroon; 60 Haiti; 28.3 Ghana). Technical considerations may limit the feasibility of certain fortification levels; concentrations up to 3 mg/g have been successfully added to bouillon in a research context ([12](#_ENREF_12)).

**References (Supplemental Material)**

1. Haile D, Luo H, Vosti SA, Dodd KW, Arnold CD, Engle-Stone R. Micronutrient Fortification of Commercially Available Biscuits Is Predicted to Have Minimal Impact on Prevalence of Inadequate Micronutrient Intakes: Modeling of National Dietary Data From Cameroon. Curr Dev Nutr. 2020 Sep;4:nzaa132.

2. Miller LV, Krebs NF, Hambidge KM. A mathematical model of zinc absorption in humans as a function of dietary zinc and phytate. J Nutr. 2007;137:135-41.

3. Miller LV, Hambidge KM, Krebs NF. Zinc Absorption Is Not Related to Dietary Phytate Intake in Infants and Young Children Based on Modeling Combined Data from Multiple Studies. J Nutr. 2015 Aug;145:1763-9.

4. Luo H, Stewart CP, Brown KH, Engle-Stone R. Predicted effects of current and potential micronutrient intervention programs on adequacy of folate and vitamin B-12 intake in a national sample of women and young children in Cameroon. The FASEB Journal 2016:891.3.

5. Doets EL, In 't Veld PH, Szczecińska A, Dhonukshe-Rutten RA, Cavelaars AE, van 't Veer P, Brzozowska A, de Groot LC. Systematic review on daily vitamin B12 losses and bioavailability for deriving recommendations on vitamin B12 intake with the factorial approach. Ann Nutr Metab. 2013;62:311-22.

6. Brown KH, Dewey K, Allen LH. Complementary feeding of young children in developing countries: a review of current scientific knowledge. Geneva: World Health Organization; 1998.

7. Brown KH, Engle-Stone R, Krebs NF, Peerson JM. Dietary intervention strategies to enhance zinc nutrition: promotion and support of breastfeeding for infants and young children. Food Nutr Bull. 2009 Mar;30:S144-71.

8. Food and Nutrition Board (FNB) Institute of Medicine (IOM). Dietary Reference Intakes for Vitamin A, Vitamin K, Arsenic, Boron, Chromium, Copper, Iodine, Iron, Manganese, Molybdenum, Nickel, Silicon, Vanadium, and Zinc. Washington D.C.: National Academy Press; 2001.

9. Hambidge KM, Miller LV, Krebs NF. Physiological requirements for zinc. Int J Vitam Nutr Res. 2011;81:72-8.

10. European Food Safety Authority. Scientific Opinion on Dietary Reference Values for zinc. EFSA Journal. 2014;12:3844.

11. Human energy requirements. Report of a Joint FAO/WHO/UNU Expert Consultation. Rome: FAO; 2001.

12. Wessells K, Kumordzie SM, Becher E, Davis JN, Nyaaba KW, Zyba S, Arnold CD, Tan X, Vosti S, et al. Acceptability of multiple micronutrient-fortified bouillon cubes among women and their households in 2 districts in the Northern Region of Ghana. Curr Dev Nutr. 2023;8.

13. Armah SM, Carriquiry A, Sullivan D, Cook JD, Reddy MB. A complete diet-based algorithm for predicting nonheme iron absorption in adults. J Nutr. 2013 Jul;143:1136-40.
